# Covalently Immobilizable Tris(Pyridino)-Crown Ether for Separation of Amines Based on Their Degree of Substitution

**DOI:** 10.3390/molecules27092838

**Published:** 2022-04-29

**Authors:** Panna Vezse, Bianka Benda, András Fekete, Ádám Golcs, Tünde Tóth, Péter Huszthy

**Affiliations:** 1 Department of Organic Chemistry and Technology, Budapest University of Technology and Economics, 1111 Budapest, Hungary; vezse.panna@edu.bme.hu (P.V.); benda.bianka@edu.bme.hu (B.B.); fekete.andras@edu.bme.hu (A.F.); toth.tunde@vbk.bme.hu (T.T.); huszthy.peter@vbk.bme.hu (P.H.); 2 Institute for Energy Security and Environmental Safety, Centre for Energy Research, 1121 Budapest, Hungary

**Keywords:** molecular recognition, crown ether, biogenic amine, separation

## Abstract

A great number of biologically active compounds contain at least one amine function. Appropriate selectivity can only be accomplished in a few cases upon the substitution of these groups, thus functionalization of amines generally results in a mixture of them. The separation of these derivatives with very similar characteristics can only be performed on a preparative scale or by applying pre-optimized HPLC methods. A tris(pyridino)-crown ether was designed and synthetized for overcoming these limitations at a molecular level. It is demonstrated, that this selector molecule is able to distinguish protonated primary, secondary and tertiary amines by the formation of reversible complexes with different stabilities. This degree of substitution-specific molecular recognition of amines opens the door to develop separation processes primarily focusing on the purification of biologically active compounds in a nanomolar scale.

## 1. Introduction

Today, the preparation of molecules, which separate various compounds is receiving increasing attention. Their application is of great importance, especially in the pharmaceutical industry [1] and environmental protection [2]. The selective complexing ability of these compounds is based on molecular recognition, by which a complex is formed with intermolecular secondary bonding forces between the host and guest molecules.

Crown ethers were the first synthetic host molecules. It was quickly realized, that their cavities can serve as binding sites for various cations, which fit into the cavities of the host molecules [3]. Regarding the molecular structure of crown ethers, the cation recognition is strongly influenced by several factors, i.e., the size of the cavity, the nature and number of donor atoms, their symmetry or the entropy conditions attributed to sterical arrangements inside the complex [4].

Amino groups are among the most frequently occurring ones of drug-like compounds, since the majority of known drugs has at least one amine unit due to the favorable pharmacokinetic properties of this functionality [5]. Biologically active synthetic compounds containing amino groups are often prepared as ammonium salts to improve their distribution in body fluids (amines usually become spontaneously protonated in living organisms due to their basicity). Moreover, the ammonium salts are typically more stable and less prone to oxidation than the free amines [6]. As a synthetic modification, *N*-alkylation or *N*-arylation of primary amines is often carried out while assembling the desired molecules. These reactions typically lack selectivity and result in a mixture of primary, secondary and tertiary amines. The separation of these amines is a recurring problem in synthetic organic chemistry. It can be traditionally solved by physical or preparative methods, i.e., fractional distillation, recrystallization or *Hinsberg*-separation when relatively large amounts of compounds are available [7]. For separating smaller quantities, HPLC is the predominant method. However, the optimization of a compound-specific method generally requires great efforts, especially in the cases of amines with highly similar polarities.

**Figure 1:**
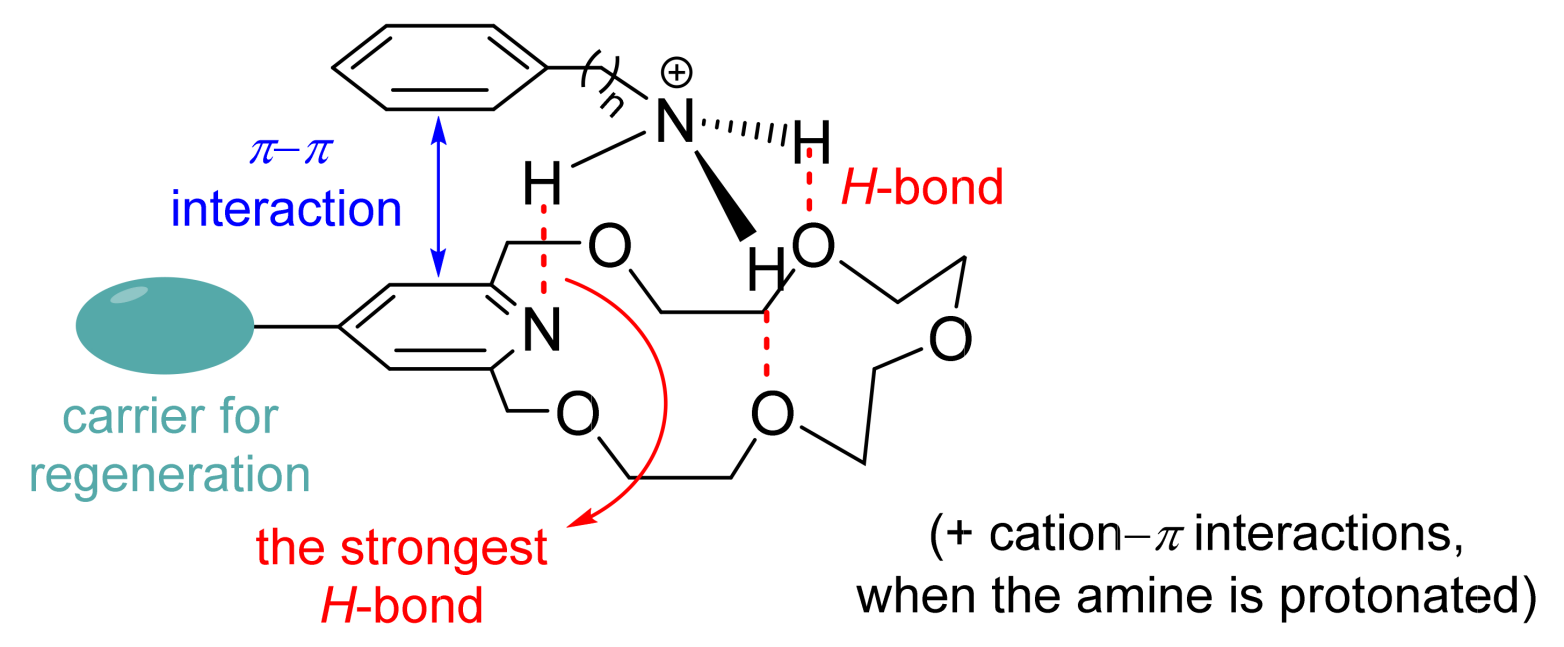
Coordination of protonated primary aralkyl amines by a covalently immobilized pyridino-18-crown-6 ether.

Macrocycles with an 18-crown-6 ether type cavity are suitable hosts for complexing protonated primary amines, since their cavity size matches the ionic radius of the primary ammonium groups (*r* = 1.4 Å and *r* = 1.42 Å, respectively), thus stable coordination can take place by accepting hydrogen bonds with the nucleophilic heteroatoms of the host [4,8] (neutral amines can also be complexed, but the complex stability is expected to be weaker in this case [9]). If a protonated primary amine guest molecule contains an aromatic unit, heterocyclic crown ethers, such as pyridino-hosts, are very advantageous as they can contribute to the effective coordination of the guest by the formation of an additional aromatic *π*-*π* interaction (Figure 1).

The strongest *H*-bond is formed between the basic pyridine-*N* and one of the ammonium protons, which is supplemented with two *H*-bonds including the *O* atoms at the alternating positions of the macroring and the rest of the ammonium protons of the aralkyl amine. The host molecules are suggested to be covalently immobilized to a carrier (e.g., nanoparticles, chromatographic stationary phase, functionalized microtiter plates, etc.) to provide regenerability in practice.

The first pyridino-crown ethers were synthetized by *Newcomb* and co-workers [10]. They found, that the stability of the complexes formed with protonated amines decreased by the number of its alkyl substituents [11]. Consequently, the strongest coordination was found with protonated primary amines. Since that time, pyridino-crown ethers were found to be suitable for separating ammonium salts from by-products [11] and for other protonated amine-selective applications including phase transport processes [12], macrocycle assisted imprinting approaches [13], drug delivery systems [14] and chemical sensing platforms [15]. Furthermore, numerous selector applications have been developed, which mainly focus on the chiral HPLC-based enantiomeric separation of optically active biogenic ammonium salts by using silica gel stationary phases containing covalently immobilized pyridino-crown ethers [16,17,18].

On the other hand, symmetric tris(pyridino)-macrocycles are barely investigated. To the best of our knowledge, only five research groups have reported this type of molecular structure before [10,11,19,20,21,22,23]. However, the properties of these hosts make them particularly suitable for the development of novel applications in the field of separation science.

We have designed a symmetric tris(pyridino)-18-crown-6 ether for molecular separation of amines based on their degree of substitution according to previously reported studies on applicability of crown ether analogues. Although the preference of the applied crown ethers toward primary amines is well-established, their expected ability for distinguishing amines based on their degree of *N*-substitution has not been exploited before. A novel applicability of this new selector molecule for the desired purpose was demonstrated by molecular recognition studies.

## 2. Results and Discussion

### 2.1. Design of the New Selector Molecule

**Figure 2:**
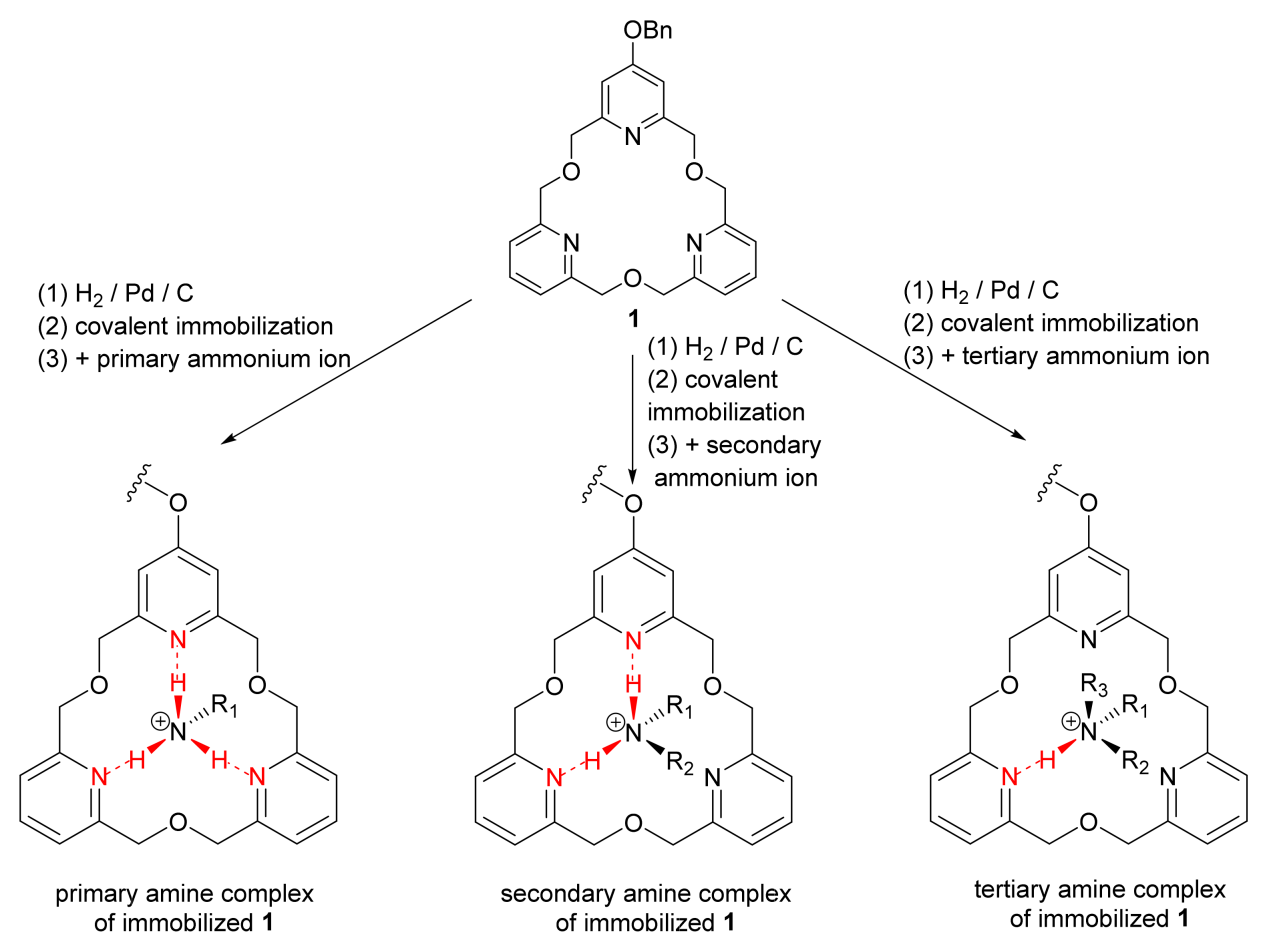
Different numbers of possible *H*-bonds of supramolecular complexes formed by the new selector molecule with protonated amines of different degrees of *N*-substitutions.

The heteroaromatic-*N* atoms of the desired selector molecule tend to coordinate soft electrophiles, such as organic ammonium cations, especially if they contain an aromatic unit. Besides the *π*-*π* interaction between the aromatic unit of the host molecule and that of the guest, the complex is stabilized by formation of intermolecular *H*-bonds. An 18-crown-6 ether type macroring was chosen as it has a ring size coinciding with the radius of the ammonium ion. The lone pairs of the nitrogen atoms as softer nucleophilic centers are most likely to be the *H*-bond-acceptors over oxygens. Since there are three symmetrically arranged heteroaromatic units, primary ammonium ions of a tetrahedral structure can be fixed by three *H*-bonds connecting to *N* atoms at alternating positions (Figure 2).

The number of possible *H*-bonds decreases with increasing degree of substitution of amines, resulting in complexes with different stabilities. The difference in complex stabilities is expected to result in selectivity. A benzyloxy group was introduced at position *4* of one of the pyridine rings as an easily transformable unit, which enables the covalent immobilization of the selector molecule after its cleavage obtaining a phenolic hydroxyl group. The covalently immobilized selector system can open a door for future applications and provides regenerability and reusability.

### 2.2. Synthesis

**Scheme 1:**
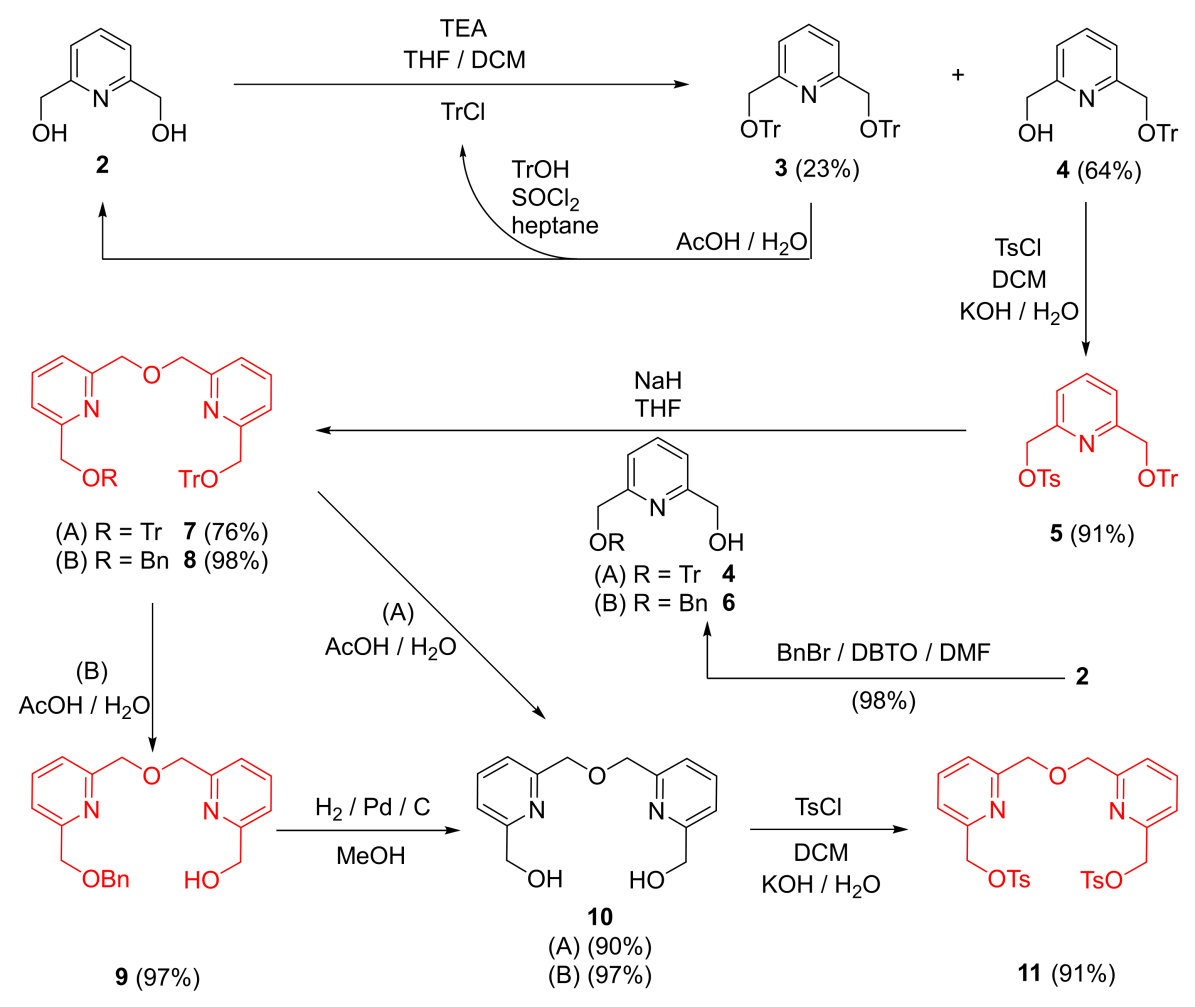
Improved synthetic procedures for the preparation of the heterocyclic key intermediates (**10**, **11**) of the new selector molecule (the new compounds are indicated in red). Compounds **2** [11], **4** [29] and **6** [29] were previously reported.

The multistep synthesis of macrocycle **1** was started with the commercially available 2,6-dimethylpyridine, which was converted to 2,6-pyridinedimethanol (**2**) according to a reported procedure [11]. Bispyridine diol **10**, the key intermediate of the synthesis, has previously been reported, but it was prepared by more complicated procedures [10,11,24,25,26,27,28]. We report here an improved and optimized synthetic pathway for obtaining diol **10**, and we also fully characterized it. Macrocycle precursors **10** and **11** were efficiently prepared as outlined in Scheme 1.

2,6-Pyridinedimethanol (**2**) was converted to its monotrityl derivative **4**. Trityl alcohol and ditrityl derivative **3** were inevitably formed in this reaction as by-products, which were recycled by reconverting them to the corresponding reagent (TrCl) and starting material **2**. Column chromatographic purification was needed to separate monotrityl derivative **4** from the crude product. The latter was then reacted with TsCl to obtain monotrityl monotosylate **5**, which was coupled with monoprotected diol **4** or **6** to gain bispyridines **7** or **8**. The coupling with monobenzylated diol **6** (procedure ‘B’) resulted in a higher yield, presumably due to less steric hindrance. Monobenzyl derivative of diol **2** (i.e., **6**) was also prepared selectively in an excellent yield using dibutyltin (IV) oxide (DBTO). The trityl groups were cleaved by using 80 *v*/*v*% aqueous solution of acetic acid, while the benzyl group of compound **9** was removed by catalytic hydrogenation. Diol **10** is soluble in water and could be efficiently purified by crystallization from a mixture of isopropyl alcohol and acetone. The hydroxyl groups of diol **10** can be tosylated to obtain ditosylate **11** with a good solubility in organic solvents.

**Scheme 2:**
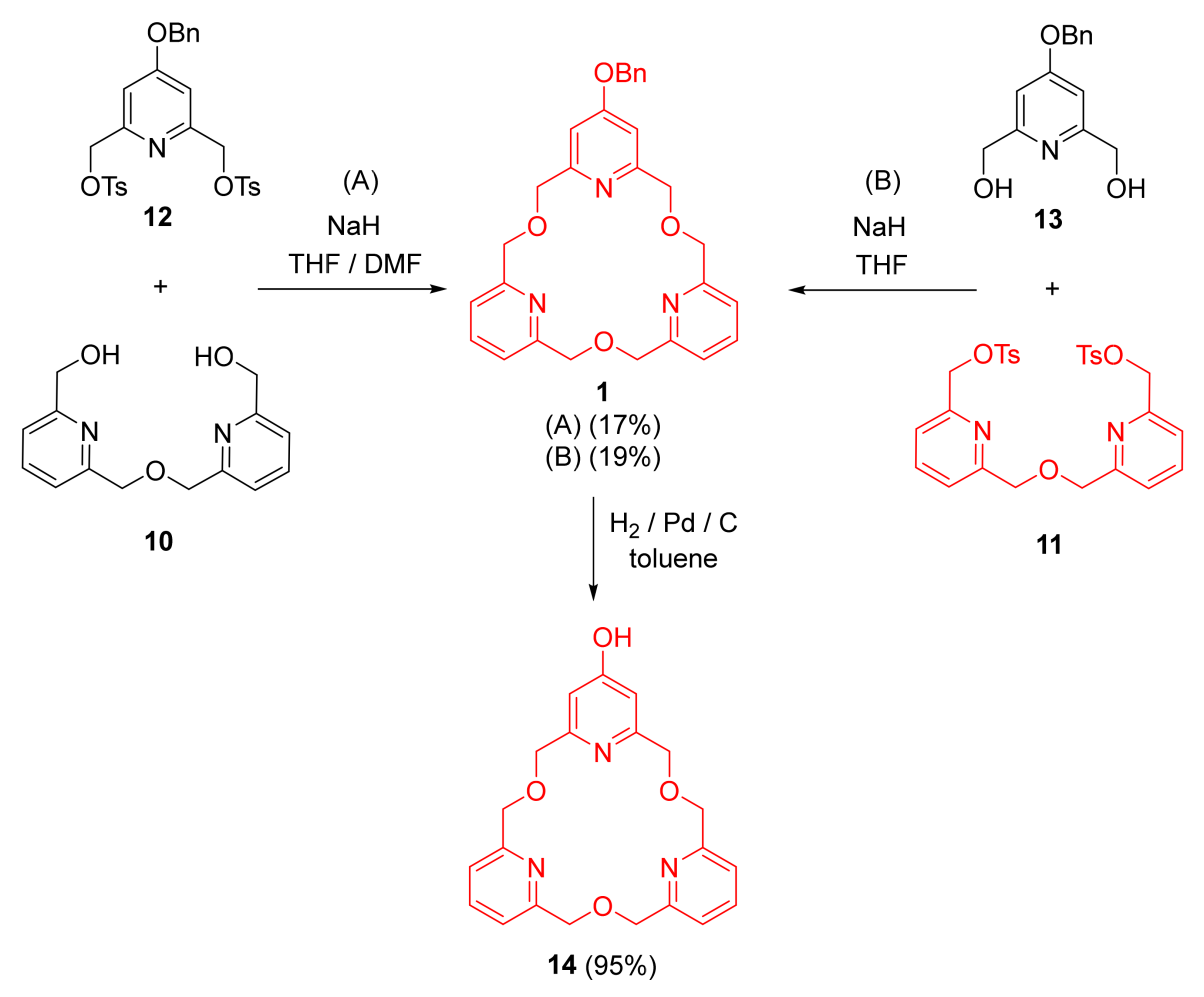
Macrocyclization of key intermediates to obtain tris(pyridino)-18-crown-6 ether **1** and its debenzylation to gain selector molecule **14** suited for covalent immobilization (the new compounds are indicated in red). Compounds **12** and **13** were previously reported [30].

The other key intermediates for macrocyclization (**12** and **13**) were prepared by a reported multistep procedure [30] starting from acetone and diethyl oxalate. The macrocyclization was carried out in two different ways as diol **10** proved to be hardly soluble in THF, which is the preferred solvent for these types of reactions using NaH (Scheme 2).

Macrocycle **1** was isolated in low yields in both cases, which are typical for these types of macrocyclizations. Since the presence of the free hydroxyl group at position *4* of one of the pyridine rings (see compound **14** in Scheme 2) can influence the complexation with ammonium ions as an additional coordinating unit, the benzyl-protected derivative of selector molecule **14**, i.e., host molecule **1**, was used for molecular recognition studies.

### 2.3. Molecular Recognition Studies

**Figure 3:**
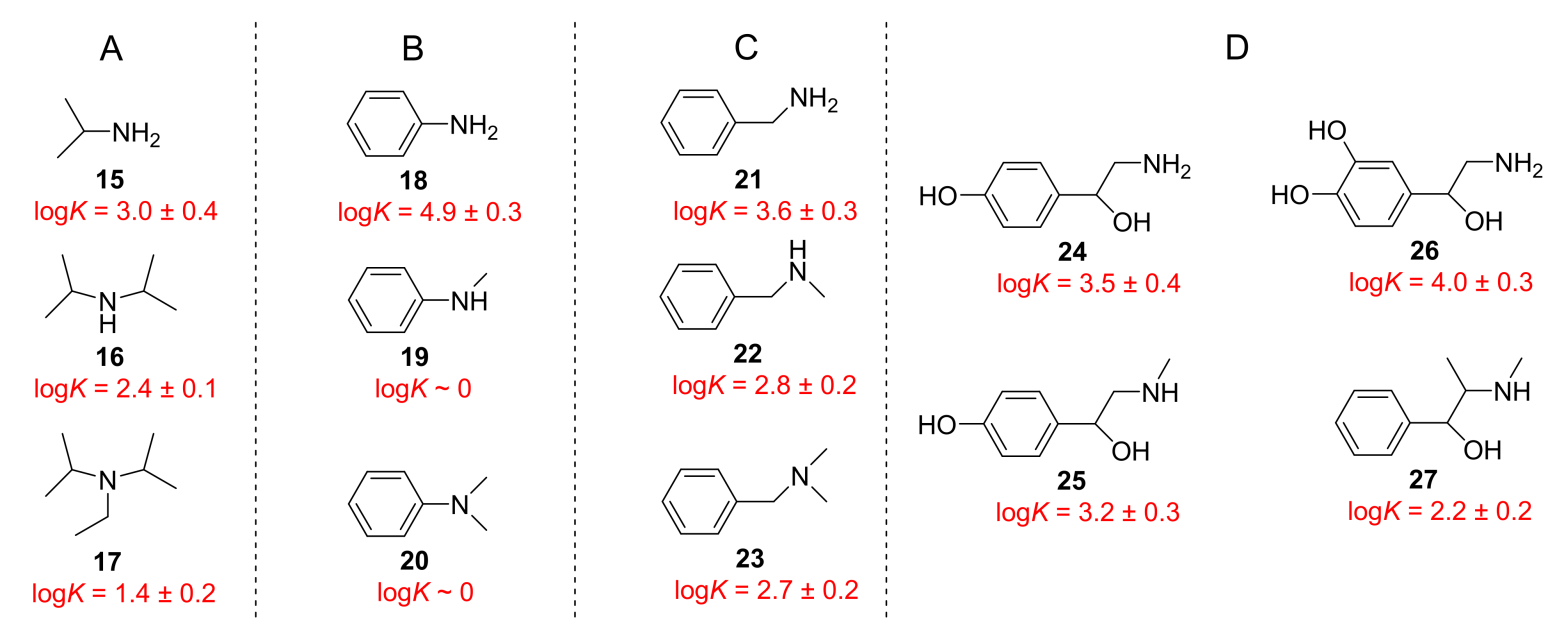
The basic classes of guest molecules including aliphatic (**A**), aromatic (**B**), aralkyl (**C**) and some bioactive (**D**) amines as model compounds and the logarithms of the spectrophotometrically determined complex stability constants (log*K* in acetonitrile for amines as hydrochloride salts) indicating the substitution-degree-based separation ability of host molecule **1**.

The molecular recognition of new host molecule **1** toward protonated aliphatic, aromatic and aralkyl amines as basic classes of the targeted guest molecules (see A, B and C in Figure 3) was studied by UV-Vis spectrophotometric titrations. These titration experiments were used to calculate the complex stability constants (*K*) of host molecule **1** with the guest molecules. All the structural classes of the selected model compounds contained three analogues of different degree of *N*-substitution. Further studies were carried out on the extendibility of the separation by investigating the discriminative power toward some bioactive small-molecule amines (see D in Figure 3), e.g., octopamine (**24**), synephrine (**25**), norepinephrine (**26**) and ephedrine (**27**) containing additional substituents in both aliphatic and aromatic units. This supplement was also useful to provide information about the influence of additional substituents on coordination of the protonated amines. The studied model amines (**15–27**) and the logarithms of the determined stability constants of the complexes are summarized in Figure 3.

The least stable complexes were obtained in the cases of aliphatic amines (A in Figure 3); probably due to the lack of *π*-*π* interaction inside the complex, which takes place in all the other cases (B–D). On the other hand, based on the differences in the complex stabilities, all derivatives can be efficiently separated from each other. The selector molecule coordinated the primary analogue (**15**) 4- and 40-fold stronger compared to the secondary (**16**) and the tertiary ones (**17**), respectively.

Among aryl (B) and aralkyl (C) amines, only the preference to primary ones is shown in the results. The secondary and tertiary amines could not be selectively recognized in this case. The highest stability of the aniline guest-(**18**)-host **1** complex might be attributed to its reduced conformational flexibility and to the presumably greater overlap of the aromatic units of host and guest upon the coordination. The stabilities of the complexes with octopamine (**24**) and synephrine (**25**) and the separabilities are very similar to those of the simpler aralkyl analogues (C). The comparison of the log*K* values in class D with those for compounds in class C suggests that the aromatic substituents certainly do not limit the substitution-degree-based recognition of amines. The example of ephedrine (**27**) shows that the methyl group at *α*-position of the amine hinders the coordination. It was expected that the groups, which are close to the ammonium unit can sterically hinder the access of the host molecule to the guests.

The determined log*K* values make the host molecule (**1**) suitable for the development of separation processes [31], while the complexes show adequate reversibility to provide regenerability without using chelating agents or other additives [32]. In practice, selectivity is prior to the stability of the complexes. The former one is characterized by the Δlog*K* values for the complexes of the differently substituted analogues. In separations, relatively small differences between complex stabilities—i.e., Δlog*K*_enantiomers_ < 0.4—can successfully be exploited [33,34,35], which is supported by numerous pyridino-crown ether-based chiral HPLC-stationary phases [16,17,18]. In these cases, selectivity is mainly provided by only sterical hindrance inside the complex. The verified selectivities in the reported novel application exceeded those of the crown ether analogues in enantioseparation. These improved selectivities can be attributed to the fact that the discriminative power is induced by the different number of the attractive intermolecular interactions. Therefore, the new selector molecule (**1**) is very promising in performing the desired target function.

## 3. Conclusions

We have successfully synthesized a new selector molecule in an optimized pathway. Spectrophotometric studies on complexation were performed in order to verify that the host molecule fulfills the desired function even in practice. The efficient discriminating ability of the selector molecule was demonstrated by determining the complex stability constants for model compounds representing different structural classes of amines.

The substitution-degree-based separation could most efficiently be performed in the case of aromatic amines as the macrocycle shows a very high preference toward the primary ones over the other analogues. However, it is not possible to separate secondary and tertiary amine guest molecules. The aralkyl amine type guests are less strongly coordinated, but their separability proved to be very similar to aromatic analogues. The selector molecule forms the least stable complexes with aliphatic amines. However, in this case, not only the primary amine guests can be distinguished from the *N*-substituted ones, but the discrimination of secondary amine guests among other analogues also occurs at the same time. According to studies on some bioactive model compounds, the increased molecular complexity—i.e., the presence of aromatic substituents or additional functional groups introduced in the aliphatic region—does not reduce applicability. It seems that only groups at *α*-position closest to the ammonium unit can limit the coordination.

As a conclusion, based on the observed several orders of magnitude differences among the complex stability constants, the proposed selector molecule is expected to be highly suitable for molecular separation of amines with different number of *N*-substituents even in practice. Moreover, the selectivity in the demonstrated and proposed function is significantly superior to that utilized for developing widespread chromatographic procedures [16,17,18] for enantioseparation of amines (Δlog*K* for enantiomers of phenylethylamine analogues is typically below 0.4 in these cases [33,34,35]). These selectivities can, for instance, be exploited to support automated syntheses, which are aimed to produce small amounts of biogenic amines for preliminary studies. Based on the reported results, the development of a high-throughput purification process using the presented macrocyclic host molecule in an immobilized form is in progress.

## 4. Experimental

### 4.1. General

Starting materials and reagents were purchased from Sigma-Aldrich Corporation (USA, owned by Merck, Darmstadt, Germany) and used without further purification unless otherwise noted. Solvents were dried and purified according to well established methods [36]. Silica Gel 60 F254 (Merck, Germany) and aluminum oxide 60 F254 neutral type E (Merck, Germany) plates were used for thin-layer chromatography (TLC). All reactions were monitored by TLC and visualized by UV-lamp. Aluminum oxide (neutral, activated, Brockman I) was used for column chromatography. Ratios of solvents for the eluents are given in volumes (mL/mL). Evaporations were carried out under reduced pressure unless otherwise stated.

The new compounds were characterized by their physical constants, such as melting point, thin-layer chromatography retention factor (*R*_f_), IR, ^1^H-NMR and ^13^C-NMR spectroscopies and HRMS spectrometry. Melting points were taken on a Boetius micro-melting point apparatus and are uncorrected. Infrared spectra were recorded on a Bruker Alpha-T FT-IR spectrometer (Bruker Corporation, Billerica, MA, USA) using KBr pastilles. ^1^H (300 MHz) and ^13^C (75 MHz) NMR spectra were recorded on a Bruker 300 Avance spectrometer (Bruker Corporation, USA). ^1^H (500 MHz) and ^13^C (125 MHz) NMR spectra were taken on a Bruker DRX-500 Avance spectrometer (Bruker Corporation, USA). HRMS analysis was carried out on a Thermo Velos Pro Orbitrap Elite (Thermo Fisher Scientific, Dreieich, Germany) system. The ionization method was ESI and was operated in positive ion mode. The protonated molecular ion peak was fragmented by CID at a normalized collision energy of 35–45%. The sample was dissolved in methanol. Data acquisition and analysis were accomplished with Xcalibur software version 2.2 (Thermo Fisher Scientific, Germany).

### 4.2. Synthesis

#### 4.2.1. Preparation of {6-[(Triphenylmethoxy)methyl]pyridin-2-yl}methyl 4-methylbenzene-1-sulfonate (**5**)

Pyridinedimethanol monotrityl ether **4** (1.00 g, 2.62 mmol, synthesized as reported [29]) was dissolved in dichloromethane (10 mL) under Ar. This solution was cooled down with an external ice-water bath, then cold aqueous solution of potassium hydroxide (10 mL, 40 *w*/*w*%) was added to it with vigorous stirring. The reaction mixture was stirred at 0 °C for 10 min. Solution of tosyl chloride (0.50 g, 2.62 mmol) in dichloromethane (10 mL) was added to it dropwise, then the mixture was stirred at room temperature for one day. Water (20 mL) and dichloromethane (10 mL) was added to the reaction mixture, the pH of the aqueous phase was adjusted to 8 with aqueous hydrochloric acid (5 *w*/*w*%), then the phases were shaken well and separated. The aqueous phase was further extracted with dichloromethane (3 × 20 mL). The combined organic phase was dried over magnesium sulfate, filtered and evaporated to gain **5** as a yellow oil. The crude product was triturated with methanol to get **5** (1.28 g, 91%) as a yellowish white solid.

M.p. = 139–140 °C. *R*_f_ = 0.76 (silica gel TLC, acetone:toluene 1:10). ^1^H-NMR (500 MHz, acetone-*d*_6_): δ [ppm]: 7.89 (t, *J* = 7.8 Hz, 1H), 7.82 (d, *J* = 8.2 Hz, 1H), 7.76 (d, *J* = 7.8 Hz, 1H), 7.56 (d, *J* = 7.8 Hz, 6H), 7.47–7.24 (m, 13H), 5.10 (s, 2H), 4.21 (s, 2H), 2.42 (s, 3H). ^13^C-NMR (75 MHz, DMSO-*d*_6_): δ [ppm]: 159.42, 158.31, 146.03, 143.87, 139.15, 138.22, 128.68, 128.62, 128.59, 127.79, 125.99, 121.81, 120.95, 87.41, 76.45, 66.76, 21.28. IR: ν_max_ [cm^−1^]: 3084, 3061, 3038, 2990, 2952, 2932, 2912, 1596, 1578, 1492, 1448, 1381, 1361, 1317, 1309, 1294, 1225, 1189, 1174, 1154, 1086, 1078, 1018, 995, 979, 955, 905, 896, 853, 825, 802, 779, 770, 763, 742, 708, 701, 694, 667, 654, 638, 631, 578, 547, 489. HRMS: *m*/*z* = [MH^+^]: 536.1818 (Calcd. for C_33_H_29_NO_4_S, 535.1817).

#### 4.2.2. Preparation of {6-[(Benzyloxy)methyl]pyridin-2-yl}methanol (**6**)

**Procedure ‘A’** (not reported in Scheme 1)

The preparation of pyridinedimethanol monobenzyl derivative **6** was reported using a two-step procedure [29]. In this case, first benzylation of pyridinedimethanol monotrityl derivative **4** (10.00 g, 26.21 mmol, BnBr/NaH/THF/DMF) was performed, then deblocking of the trityl group was carried out by aqueous acetic acid. These reactions were done in the same way as described by *Tsukube* and co-workers except that column chromatography was omitted. The crude monobenzyl-monotrityl derivative of pyridinedimethanol **2** ([29], it is not shown in Scheme 1) was purified by trituration with methanol to obtain the pure product (12.11 g, 98%) as white crystals. The trityl group was also removed by modifying the reported procedure [29] using a mixture of dichloromethane:methanol:concentrated hydrochloric acid (37 *w*/*w*%) (1:1:1, 100 mL/1.00 g starting material) at room temperature. The latter reaction mixture was worked up and the crude product was purified as reported [29] to get **6** as a colorless oil. The products were identical in every aspect with those reported [29].

**Procedure ‘B’**

Selective monobenzylation was also carried out in an earlier unreported one-step procedure. Starting material **2** (10.00 g, 71.86 mmol) and dibutyltin (IV) oxide (17.88 g, 71.86 mmol) were dissolved in *N*,*N*-dimethylformamide (300 mL) under Ar. This mixture was stirred at room temperature for 1 h, then freshly distilled benzyl bromide (9.40 mL, 79.05 mmol) was added to it dropwise. The temperature of the reaction mixture was raised to 80 °C and it was stirred at this temperature for 2 d. The solvent was removed and the residue was taken up in water (200 mL) and ethyl acetate (200 mL). The phases were shaken well and separated. The aqueous phase was extracted with ethyl acetate (3 × 200 mL). The combined organic phase was dried over magnesium sulfate, filtered and evaporated to give **6** (16.15 g, 98%) as a colorless oil. The product did not need purification and it was identical in every aspect with that reported [29].

#### 4.2.3. Preparation of 2-[(Triphenylmethoxy)methyl]-6-[({6-[(triphenylmethoxy)methyl]pyridin-2-yl}methoxy)methyl]pyridine (**7**)

Sodium hydride (0.79 g, 19.67 mmol, 60 *w*/*w*% dispersion in mineral oil) was suspended in dry tetrahydrofuran (20 mL) under Ar at room temperature. Solution of **4** (5.00 g, 13.11 mmol) in tetrahydrofuran (50 mL) was added dropwise to the stirred suspension of sodium hydride. The resulting mixture was refluxed for 30 min. The temperature of the reaction mixture was set to 0 °C with an external ice-water bath, then a solution of **5** (7.02 g, 13.11 mmol) in tetrahydrofuran (50 mL) was added dropwise and it was stirred for 1 h at this temperature. The temperature of the reaction mixture was raised to boiling point and it was refluxed for 24 h. The solvent was evaporated and the residue was mixed with cold water (100 mL), then this aqueous mixture was extracted with chloroform (3 × 100 mL). The combined organic phase was dried over magnesium sulfate, filtered and evaporated. The crude product was recrystallized from ethyl acetate to gain **7** (7.42 g, 76%) as a yellowish white solid.

M.p. = 60–64 °C. *R*_f_ = 0.69 (silica gel TLC, acetone:toluene 1:10). ^1^H-NMR (500 MHz, acetone-*d*_6_): δ [ppm]: 7.83 (td, *J* = 7.8, 2.0 Hz, 2H), 7.68 (d, *J* = 7.7 Hz, 2H), 7.55 (d, *J* = 7.4 Hz, 12H), 7.43 (d, *J* = 7.7 Hz, 2H), 7.34 (t, *J* = 7.4 Hz, 12H), 7.26 (t, *J* = 7.4 Hz, 6H), 4.63 (s, 4H), 4.25 (s, 4H). ^13^C-NMR (125 MHz, acetone-*d*_6_): δ [ppm]: 158.18, 157.83, 144.03, 137.27, 128.58, 127.91, 127.17, 119.63, 119.36, 87.15, 73.39, 66.94. IR: ν_max_ [cm^−1^]: 3085, 3058, 3031, 2954, 2924, 2856, 2314, 1959, 1897, 1815, 1776, 1722, 1646, 1594, 1578, 1490, 1448, 1398, 1359, 1277, 1219, 1178, 1153, 1096, 1032, 986, 898, 848, 778, 762, 746, 706, 632, 572, 537, 458. HRMS: *m*/*z* = [MH^+^]: 745.3346 (Calcd. for C_52_H_44_N_2_O_3_, 744.3352).

#### 4.2.4. Preparation of 2-[(Benzyloxy)methyl]-6-[({6-[(triphenylmethoxy)methyl]pyridin-2-yl}methoxy)methyl]pyridine (**8**)

Sodium hydride (1.31 g, 32.72 mmol, 60 *w*/*w*% dispersion in mineral oil) was suspended in dry tetrahydrofuran (20 mL) under Ar at room temperature. Solution of **6** (5.00 g, 21.81 mmol) in tetrahydrofuran (50 mL) was added dropwise to the stirred suspension of sodium hydride. The resulting mixture was refluxed for 30 min. The temperature of the reaction mixture was set to 0 °C with an external ice-water bath, then a solution of **5** (11.68 g, 21.81 mmol) in tetrahydrofuran (50 mL) was added dropwise and it was stirred for 1 h at this temperature. The temperature of the reaction mixture was raised to boiling point and it was refluxed for 24 h. The solvent was evaporated and the residue was mixed with cold water (100 mL), then this aqueous mixture was extracted with chloroform (3 × 100 mL). The combined organic phase was dried over magnesium sulfate, filtered and evaporated. The crude product was recrystallized from ethyl acetate to gain **8** (12.67 g, 98%) as a white solid.

M.p. = 73–74 °C. *R*_f_ = 0.50 (silica gel TLC, ethanol:toluene 1:10). ^1^H-NMR (500 MHz, DMSO-*d*_6_): δ [ppm]: 7.87 (t, *J* = 7.8 Hz, 1H), 7.78 (t, *J* = 7.8 Hz, 1H), 7.61 (d, *J* = 7.8 Hz, 1H), 7.45–7.41 (m, 7H), 7.40–7.38 (m, 1H), 7.35–7.32 (m, 10H), 7.28–7.23 (m, 5H), 4.61 (s, 2H), 4.58 (s, 2H), 4.56 (s, 2H), 4.55 (s, 2H), 4.09 (s, 2H). ^13^C-NMR (125 MHz, DMSO-*d*_6_): δ [ppm]: 158.05, 157.94, 157.77, 157.74, 143.96, 138.60, 138.20, 137.92, 128.74, 128.63, 128.52, 128.05, 127.98, 127.67, 120.47, 119.88, 87.15, 73.42, 73.36, 72.92, 72.38, 66.96. IR: ν_max_ [cm^−1^]: 3085, 3059, 3031, 2914, 2854, 1593, 1578, 1490, 1447, 1358, 1215, 1152, 1094, 1030, 987, 898, 745, 696, 632, 572. HRMS: *m*/*z* = [MH^+^]: 593.2730 (Calcd. for C_40_H_36_N_2_O_3_, 592.2726).

#### 4.2.5. General Procedure for Removing Trityl Protective Group

A trityl derivative was dissolved in aqueous acetic acid (50 mL/1.00 g starting material, 80 *v*/*v*%) under Ar. This solution was stirred at 40 °C for 2 d, then evaporated. The residue was mixed with cold water (100 mL/1.00 g starting material), neutralized with saturated aqueous solution of sodium carbonate and extracted with diethyl ether (5 × 50 mL/1.00 g starting material). The combined organic phase was dried over magnesium sulfate, filtered and evaporated.

#### 4.2.6. Preparation of {6-[({6-[(Benzyloxy)methyl]pyridin-2-yl}methoxy)methyl]pyridin-2-yl}methanol (**9**)

The trityl protective group of **8** (5.00 g, 8.44 mmol) was removed according to the general procedure reported in Section 4.2.5. The crude product was recrystallized from ethyl acetate to give **9** (2.87 g, 97%) as a white solid.

M.p. = 84–85 °C. *R*_f_ = 0.20 (silica gel TLC, ethanol:toluene 1:10). ^1^H-NMR (500 MHz, methanol-*d*_4_): δ [ppm]: 7.81 (td, *J* = 7.8, 4.0 Hz, 2H), 7.51–7.40 (m, 4H), 7.38–7.17 (m, 5H), 4.70 (s, 4H), 4.66 (s, 2H), 4.61 (s, 2H), 4.61 (s, 2H). ^13^C-NMR (125 MHz, methanol-*d*_4_): δ [ppm]: 164.51, 161.73, 161.30, 161.02, 141.87, 141.77, 131.98, 131.51, 131.36, 124.47, 124.44, 124.01, 123.30, 76.78, 76.73, 76.50, 76.01, 68.04. IR: ν_max_ [cm^−1^]: 3339, 3086, 3062, 3027, 2918, 2883, 2850, 1596, 1577, 1449, 1351, 1257, 1209, 1121, 1031, 1002, 990, 900, 800, 782, 727, 693, 627, 560, 504, 459. HRMS: *m*/*z* = [MH^+^]: 351.1634 (Calcd. for C_21_H_22_N_2_O_3_, 350.1630).

#### 4.2.7. Preparation of (6-{[(6-{[(4-Methylbenzenesulfonyl)oxy]methyl}pyridin-2-yl)methoxy]methyl}pyridin-2-yl)methyl 4-methylbenzene-1-sulfonate (**11**)

The synthesis of diol **10**, which is the precursor of ditosylate **11** was carried out in two different ways (procedure ‘A’ starting from **7** and procedure ‘B’ starting from **8**).

**Procedure ‘A’**

The trityl protective groups of **7** (5.00 g, 6.71 mmol) were removed according to the general procedure reported in Section 4.2.5. The crude product was recrystallized from isopropyl alcohol-acetone solvent mixture to give **10** (1.57 g, 90%) as a white solid.

M.p. = 103–104 °C. *R*_f_ = 0.29 (silica gel TLC, methanol:toluene 1:4). ^1^H-NMR (500 MHz, methanol-*d*_4_): δ [ppm]: 7.87 (t, *J* = 7.8 Hz, 2H), 7.44 (d, *J* = 7.8 Hz, 4H), 4.83 (s, 4H), 4.80 (s, 4H). ^13^C-NMR (125 MHz, acetone-*d*_4_): δ [ppm]: 159.94, 156.60, 138.18, 119.99, 119.50, 72.70, 63.84. IR: ν_max_ [cm^−1^]: 3357, 3134, 3123, 3002, 2917, 2872, 1597, 1577, 1466, 1458, 1443, 1411, 1396, 1354, 1344, 1237, 1213, 1139, 1095, 1078, 1054, 1024, 1000, 962, 801, 790, 773, 640, 608, 543. HRMS: *m*/*z* = [MH^+^]: 261.1162 (Calcd. for C_14_H_16_N_2_O_3_, 260.1161).

**Procedure ‘B’**

A solution of **9** (5.00 g, 14.27 mmol) in methanol (100 mL) was hydrogenated in the presence of Pd/C catalyst (0.50 g, Pd/charcoal; activated, 10% Pd) with a catalytic amount of perchloric acid at 40 °C. After 5 h the reaction was complete. The catalyst was filtered off and washed with methanol (2 × 50 mL). The filtrate and washings were evaporated to give **10** (3.60 g, 97%) as a white solid. This product did not need purification and it was identical in every aspect with that described above.

Diol **10** (3.00 g, 11.53 mmol) was dissolved in dichloromethane (20 mL) under Ar. The temperature of this solution was set to 0 °C with an external ice-water bath, then a cold aqueous solution of potassium hydroxide (20 mL, 40 *w*/*w*%) was added to it. The resulting mixture was stirred at 0 °C for 10 min. A solution of tosyl chloride (4.84 g, 25.37 mmol) in dichloromethane (20 mL) was added dropwise, then the mixture was stirred at room temperature for one day. Water (30 mL) and dichloromethane (10 mL) were added to the reaction mixture, the pH of the aqueous phase was adjusted to 8 with aqueous hydrochloric acid (5 *w*/*w*%), then the phases were shaken well and separated. The aqueous phase was further extracted with dichloromethane (3 × 20 mL). The combined organic phase was dried over magnesium sulfate, filtered and evaporated. The crude product was recrystallized from methanol-dichloromethane solvent mixture to get **11** (5.97 g, 91%) as a yellowish white solid.

M.p. = 128–129 °C. *R*_f_ = 0.30 (silica gel TLC, ethanol:toluene 1:10). ^1^H-NMR (500 MHz, acetone-*d*_6_): δ [ppm]: 7.89–7.81 (m, 6H), 7.52 (d, *J* = 7.8 Hz, 2H), 7.47 (d, *J* = 8.0 Hz, 4H), 7.35 (d, *J* = 7.7 Hz, 2H), 5.16 (s, 4H), 4.66 (s, 4H), 2.45 (s, 6H). ^13^C-NMR (125 MHz, acetone-*d*_6_): δ [ppm]: 158.43, 153.07, 145.11, 137.61, 133.35, 129.97, 127.94, 121.06, 120.97, 73.17, 72.14, 20.65. IR: ν_max_ [cm^−1^]: 3064, 3003, 2917, 2872, 2850, 2728, 2629, 2172, 2019, 1905, 1741, 1598, 1577, 1467, 1444, 1422, 1383, 1385, 1344, 1328, 1216, 1140, 1095, 1084, 1054, 1023, 993, 982, 898, 814, 802, 790, 774, 640, 610, 542, 451. HRMS: *m*/*z* = [MH^+^]: 569.1419 (Calcd. for C_28_H_28_N_2_O_7_S_2_, 568.1338).

#### 4.2.8. Preparation of 23-(Benzyloxy)-3,11,19-trioxa-25,26,27-triazatetracyclo[19.3.1.1^5,9^.1^13,17^]heptacosa-1(25),5,7,9(27),13(26),14,16,21,23-nonaene (**1**)

**Procedure ‘A’**

Sodium hydride (1.38 g, 34.59 mmol, 60 *w*/*w*% dispersion in mineral oil) was suspended in dry tetrahydrofuran (30 mL) under Ar at 0 °C. A solution of **10** (3.00 g, 11.53 mmol) in dry *N*,*N*-dimethylformamide (50 mL) was added dropwise to this stirred suspension. The resulting mixture was stirred at 0 °C for 2 h, then a solution of **12** [30] (6.38 g, 11.53 mmol) in tetrahydrofuran and *N*,*N*-dimethylformamide (400 mL, 1:1) was added dropwise and the reaction mixture was stirred for 2 h. The temperature of the reaction mixture was raised to 50 °C and it was stirred for 14 d. The solvents were evaporated and the residue was taken up in cold water and ethyl acetate (400 mL, 1:1). The phases were shaken well and separated. The aqueous phase was extracted with ethyl acetate (3 × 200 mL). The combined organic phase was dried over magnesium sulfate, filtered and evaporated. The crude product was purified by column chromatography on neutral Al_2_O_3_ using a mixture of ethanol and toluene (1:10) as an eluent to gain **1** (920 mg, 17%) as a white solid.

M.p. = 96 °C. *R*_f_ = 0.52 (silica gel TLC, methanol:toluene 1:4). ^1^H-NMR (500 MHz, acetone-*d*_6_): δ [ppm]: 7.65 (t, *J* = 7.7 Hz, 2H), 7.49 (d, *J* = 7.1 Hz, 2H), 7.44 (t, *J* = 7.4 Hz, 3H), 7.25 (d, *J* = 7.7 Hz, 4H), 6.82 (s, 2H), 5.13 (s, 2H), 4.63 (s, 4H), 4.61 (s, 4H), 4.58 (s, 4H). ^13^C-NMR (125 MHz, acetone-*d*_6_): δ [ppm]: 205.23, 165.79, 159.70, 157.76, 136.63, 128.48, 127.99, 127.67, 120.49, 120.29, 106.78, 72.83, 72.62, 72.52, 69.40. IR: ν_max_ [cm^−1^]: 3064, 3028, 2961, 2923, 2889, 2867, 2850, 2709, 2475, 1597, 1576, 1496, 1456, 1424, 1401, 1379, 1367, 1323, 1251, 1233, 1221, 1155, 1147, 1108, 1094, 1046, 1041, 1029, 1015, 994, 962, 918, 860, 853, 839, 780, 766, 754, 720, 702, 696, 609, 598, 526. HRMS: *m*/*z* = [MH^+^]: 470.2092 (Calcd. for C_28_H_27_N_3_O_4_, 469.2002).

**Procedure ‘B’**

Sodium hydride (0.21 g, 5.28 mmol, 60 *w*/*w*% dispersion in mineral oil) was suspended in dry tetrahydrofuran (10 mL) under Ar at 0 °C. A solution of **13** [30] (0.43 g, 1.76 mmol) in dry tetrahydrofuran (50 mL) was added dropwise to this stirred suspension. The resulting mixture was stirred at 0 °C for 2 h, then a solution of **11** (1.00 g, 1.76 mmol) in dry tetrahydrofuran (200 mL) was added dropwise to it. Stirring was continued at this temperature for 2 h. The temperature of the reaction mixture was raised to 50 °C and it was stirred for 14 d. The volatile components were evaporated. The residue was taken up in cold water and ethyl acetate (300 mL, 1:1). The phases were shaken well and separated. The aqueous phase was extracted with ethyl acetate (3 × 200 mL). The combined organic phase was dried over magnesium sulfate, filtered and evaporated. The crude product was purified by column chromatography on neutral Al_2_O_3_ using a mixture of ethanol and toluene (1:10) as an eluent to gain **1** (157 mg, 19%) as a white solid. The product was identical in every aspect with that prepared according to procedure ‘A’.

#### 4.2.9. Preparation of 3,11,19-Trioxa-25,26,27-triazatetracyclo[19.3.1.1^5,9^.1^13,17^]heptacosa-1(24),5,7,9(27),13,15,17(26),21(25),22-nonaen-7-ol (**14**)

A solution of **1** (0.50 g, 1.06 mmol) in toluene (100 mL) was hydrogenated in the presence of Pd/C catalyst (0.10 g, Pd/charcoal; activated, 10% Pd) at 50 °C. After 8 h the reaction was complete. The catalyst was filtered off and washed with methanol (2 × 50 mL). The filtrate and washings were evaporated. The crude product was recrystallized from ethanol to give **14** (382 mg, 95%) as a white solid.

M.p. = 89 °C. *R*_f_ = 0.46 (silica gel TLC, methanol:toluene 1:4). ^1^H-NMR (500 MHz, CDCl_3_): δ [ppm]: 7.68 (t, *J* = 7.8 Hz, 2H), 7.32–7.27 (m, 4H), 7.18–7.11 (m, 2H), 4.73 (s, 4H), 4.68 (s, 4H), 4.64 (s, 4H). ^13^C-NMR (125 MHz, CDCl_3_): δ [ppm]: 158.39, 157.56, 144.03, 137.92, 137.36, 128.48, 119.93, 119.14, 72.96, 72.85, 63.99. IR: ν_max_ [cm^−1^]: 3330, 3064, 3028, 2961, 2923, 2889, 2867, 2850, 1597, 1576, 1496, 1456, 1379, 1367, 1323, 1251, 1221, 1155, 1108, 1094, 1041, 1015, 994, 962, 918, 860, 853, 839, 780, 766, 754, 696, 598, 526. HRMS: *m*/*z* = [MH^+^]: 380.1535 (Calcd. for C_21_H_21_N_3_O_4_, 379.1532).

### 4.3. Molecular Recognition Studies

UV-Vis spectra were recorded on a UNICAM UV4-100 spectrophotometer controlled by VIZION 3.4 software (ATI UNICAM, Hatley Saint George, UK). Quartz cuvettes with a path length of 1 cm were used in all cases. Spectroscopic measurements were carried out at room temperature (25 ± 1 °C). During spectrophotometric titrations, the acetonitrile solutions of hydrochloride salts of amines were added with a *Hamilton*-syringe to the acetonitrile solution of host **1**. The reported spectra were corrected in each case with the background signal of the added solutions and concentration values were also corrected corresponding to the caused dilution. OriginPro 8.6 (OriginLab Corp., Northampton, MA, USA) software was used for evaluation and visualization of the spectroscopic results. The stability constants of the complexes were determined by global non-linear regression analysis on experimental titration data. Fitting of mathematical models was carried out according to a reported procedure [37]. Additional information regarding spectrophotometric studies and regression analyses can be found in the Supplementary Materials.

## Supplementary Materials

The following supporting information can be downloaded at: https://www.mdpi.com/article/10.3390/molecules27092838/s1, Figure S1: ^1^H-NMR spectrum of **5** (acetone-*d*_6_); Figure S2: ^13^C-NMR spectrum of **5** (CDCl_3_); Figure S3: ^1^H-NMR spectrum of **7** (acetone-*d*_6_); Figure S4: ^13^C-NMR spectrum of **7** (acetone-*d*_6_); Figure S5: ^1^H-NMR spectrum of **8** (DMSO-*d*_6_); Figure S6: ^13^C-NMR spectrum of **8** (DMSO-*d*_6_); Figure S7: ^1^H-NMR spectrum of **9** (methanol-*d*_4_); Figure S8: ^13^C-NMR spectrum of **9** (CDCl_3_); Figure S9: ^1^H-NMR spectrum of **11** (acetone-*d*_6_); Figure S10: ^13^C-NMR spectrum of **11** (acetone-*d*_6_); Figure S11: ^1^H-NMR spectrum of **1** (acetone-*d*_6_); Figure S12: ^13^C-NMR spectrum of **1** (acetone-*d*_6_); Figure S13: ^1^H-NMR spectrum of **14** (CDCl_3_); Figure S14: ^13^C-NMR spectrum of **14** (CDCl_3_); Figure S15: ^1^H-NMR spectrum of **10** (methanol-*d*_4_); Figure S16: ^13^C-NMR spectrum of **10** (methanol-*d*_4_); Figure S17: Series of absorption spectra for titrating macrocycle **1** with protonated amine **15** in acetonitrile (c_macrocycle_ = 5 × 10^−7^ mol·L^−1^); Figure S18: The globally fitted nonlinear regression curve for determining log*K* value based on titrating macrocycle **1** with protonated amine **15** in acetonitrile (c_macrocycle_ = 5 × 10^−7^ mol·L^−1^); Figure S19: Series of absorption spectra for titrating macrocycle **1** with protonated amine **16** in acetonitrile (c_macrocycle_ = 5 × 10^−7^ mol·L^−1^); Figure S20: The globally fitted nonlinear regression curve for determining log*K* value based on titrating macrocycle **1** with protonated amine **16** in acetonitrile (c_macrocycle_ = 5 × 10^−7^ mol·L^−1^); Figure S21: Series of absorption spectra for titrating macrocycle **1** with protonated amine **17** in acetonitrile (c_macrocycle_ = 5 × 10^−7^ mol·L^−1^); Figure S22: The globally fitted nonlinear regression curve for determining log*K* value based on titrating macrocycle **1** with protonated amine **17** in acetonitrile (c_macrocycle_ = 5 × 10^−7^ mol·L^−1^); Figure S23: Series of absorption spectra for titrating macrocycle **1** with protonated amine **18** in acetonitrile (c_macrocycle_ = 5 × 10^−8^ mol·L^−1^); Figure S24: The globally fitted nonlinear regression curve for determining log*K* value based on titrating macrocycle **1** with protonated amine **18** in acetonitrile (c_macrocycle_ = 5 × 10^−8^ mol·L^−1^); Figure S25: Series of absorption spectra for titrating macrocycle **1** with protonated amine **21** in acetonitrile (c_macrocycle_ = 5 × 10^−8^ mol·L^−1^); Figure S26: The globally fitted nonlinear regression curve for determining log*K* value based on titrating macrocycle **1** with protonated amine **21** in acetonitrile (c_macrocycle_ = 5 × 10^−8^ mol·L^−1^); Figure S27: Series of absorption spectra for titrating macrocycle **1** with protonated amine **22** in acetonitrile (c_macrocycle_ = 5 × 10^−8^ mol·L^−1^); Figure S28: The globally fitted nonlinear regression curve for determining log*K* value based on titrating macrocycle **1** with protonated amine **22** in acetonitrile (c_macrocycle_ = 5 × 10^−8^ mol·L^−1^); Figure S29: Series of absorption spectra for titrating macrocycle **1** with protonated amine **23** in acetonitrile (c_macrocycle_ = 5 × 10^−8^ mol·L^−1^); Figure S30: The globally fitted nonlinear regression curve for determining log*K* value based on titrating macrocycle **1** with protonated amine **23** in acetonitrile (c_macrocycle_ = 5 × 10^−8^ mol·L^−1^); Figure S31: Series of absorption spectra for titrating macrocycle **1** with protonated amine **24** in acetonitrile (c_macrocycle_ = 5 × 10^−8^ mol·L^−1^); Figure S32: The globally fitted nonlinear regression curve for determining log*K* value based on titrating macrocycle **1** with protonated amine **24** in acetonitrile (c_macrocycle_ = 5 × 10^−8^ mol·L^−1^); Figure S33: Series of absorption spectra for titrating macrocycle **1** with protonated amine **25** in acetonitrile (c_macrocycle_ = 5 × 10^−8^ mol·L^−1^); Figure S34: The globally fitted nonlinear regression curve for determining log*K* value based on titrating macrocycle **1** with protonated amine **25** in acetonitrile (c_macrocycle_ = 5 × 10^−8^ mol·L^−1^); Figure S35: Series of absorption spectra for titrating macrocycle **1** with protonated amine **26** in acetonitrile (c_macrocycle_ = 5 × 10^−8^ mol·L^−1^); Figure S36: The globally fitted nonlinear regression curve for determining log*K* value based on titrating macrocycle **1** with protonated amine **26** in acetonitrile (c_macrocycle_ = 5 × 10^−8^ mol·L^−1^); Figure S37: Series of absorption spectra for titrating macrocycle **1** with protonated amine **27** in acetonitrile (c_macrocycle_ = 5 × 10^−8^ mol·L^−1^); Figure S38: The globally fitted nonlinear regression curve for determining log*K* value based on titrating macrocycle **1** with protonated amine **27** in acetonitrile (c_macrocycle_ = 5 × 10^−8^ mol·L^−1^).
