# Supplementary material for: Covalently Immobilizable Tris(Pyridino)-Crown Ether for Separation of Amines Based on Their Degree of Substitution
Source: Molecules. 2022 Apr 29;27(9):2838. doi: 10.3390/molecules27092838 (PMC9103866; doi:10.3390/molecules27092838)
Supplement: Supplementary file 1 [file molecules-27-02838-s001.zip › molecules-1686919-supplementary.pdf]

Supplementary Material

Covalently immobilizable tris(pyridino)-crown ether for separation of amines based on their degree of substitution

Panna Vezse,[a] Bianka Benda,[a] András Fekete,[a]
Ádám Golcs,*[a] Tünde Tóth,[a,b] Péter Huszthy[a]

[a] Department of Organic Chemistry and Technology, Budapest University of Technology and Economics, Szent Gellért tér 4., H-1111 Budapest, Hungary
[b] Institute for Energy Security and Environmental Safety, Centre for Energy Research, Konkoly-Thege Miklós út 29-33., H-1121 Budapest, Hungary
*Corresponding author. Tel.: +36 20 622 6164, E-mail address: golcs.adam@edu.bme.hu

Contents

1. ^{1}H-NMR and ^{13}C-NMR spectra of the new compounds
2. ^{1}H-NMR and ^{13}C-NMR spectra of the previously reported partially characterized compound **10**
3. Spectrophotometric investigation of the new crown ether (**1**)

1. ^{1}H-NMR and ^{13}C-NMR spectra of the new compounds

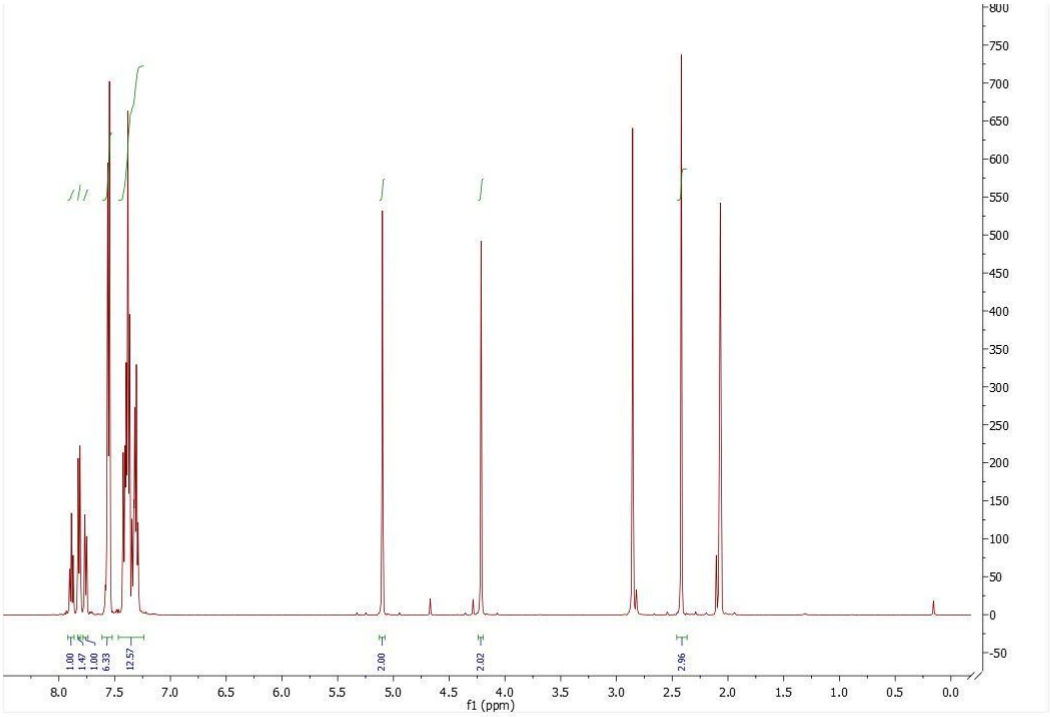

Figure S1. ^{1}H-NMR spectrum of **5** (acetone-d_6)

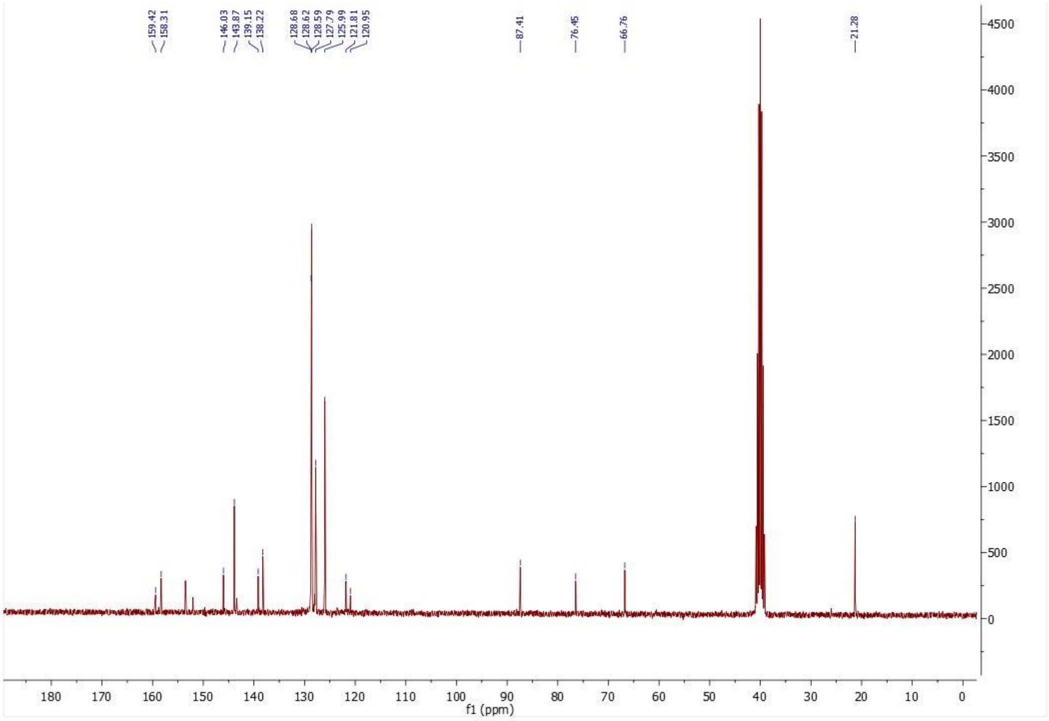

Figure S2. ^{13}C-NMR spectrum of **5** ($CDCl_3$)

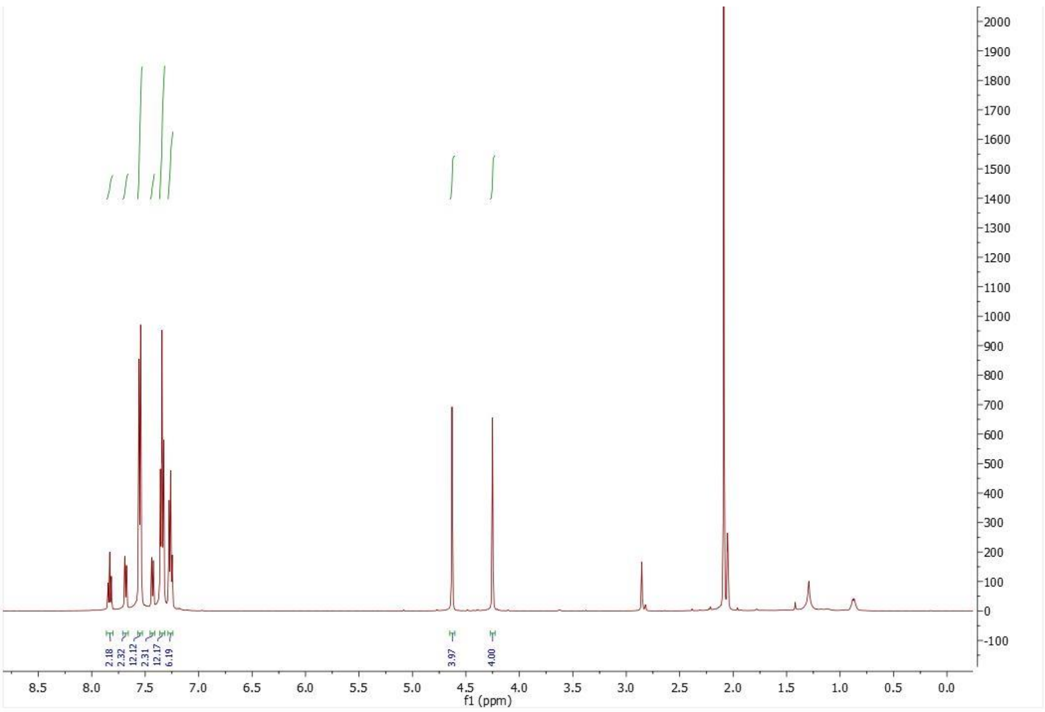

Figure S3. ^{1}H-NMR spectrum of **7** (acetone-d_6)

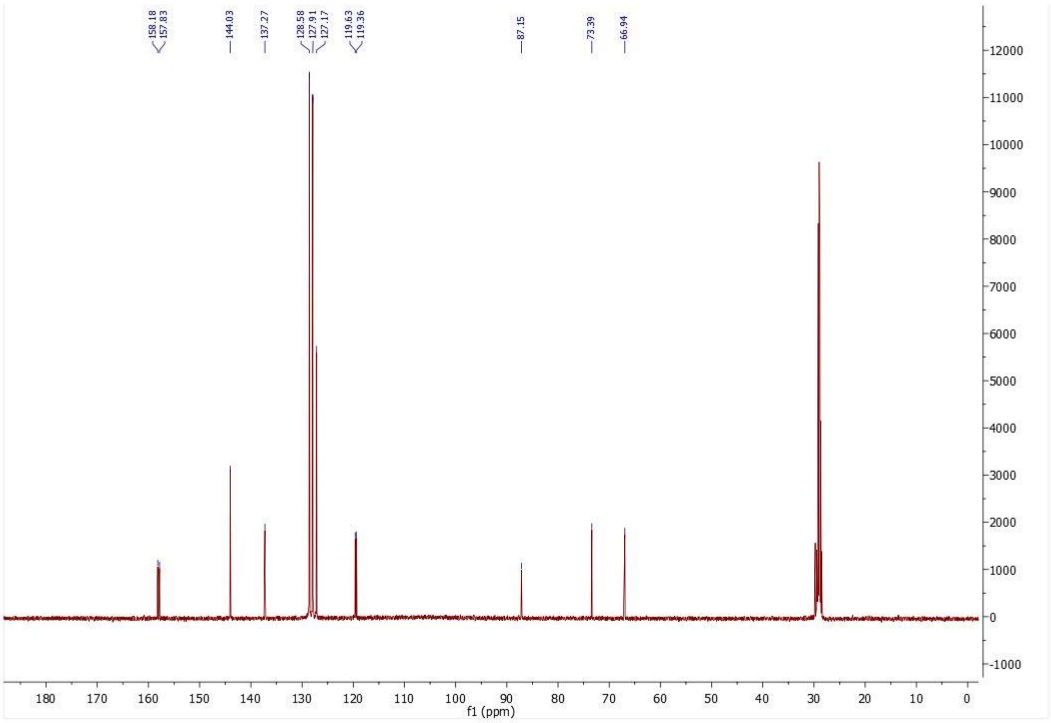

Figure S4. ^{13}C-NMR spectrum of **7** (acetone-d_6)

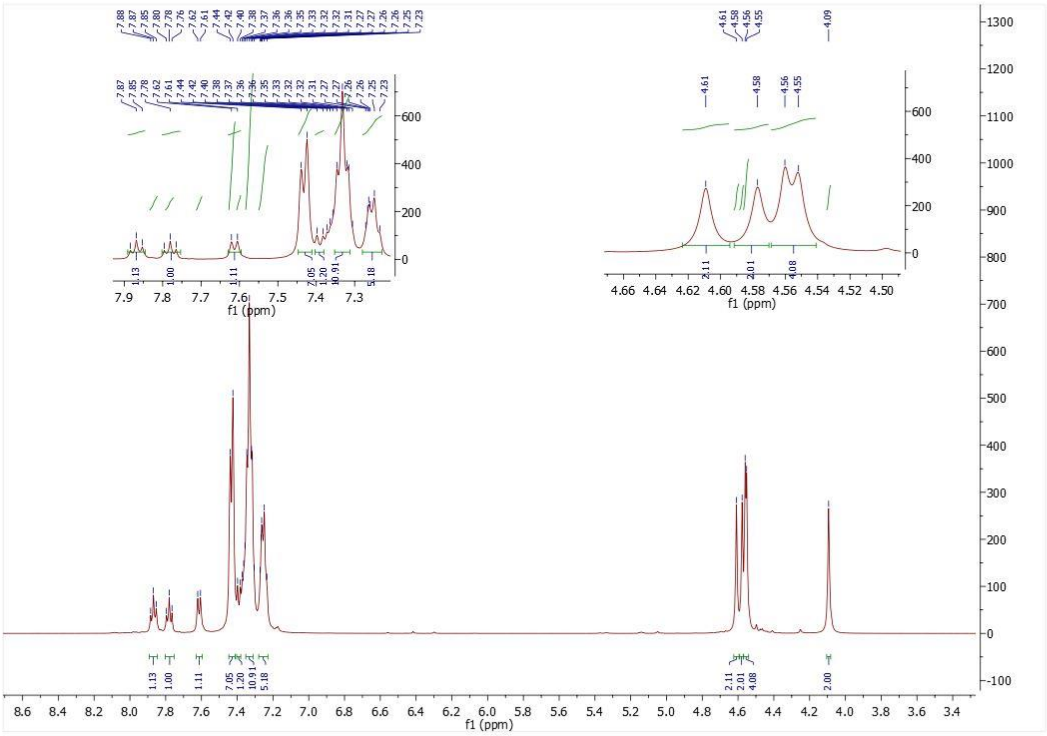

Figure S5. ^{1}H-NMR spectrum of **8** (DMSO-d_6)

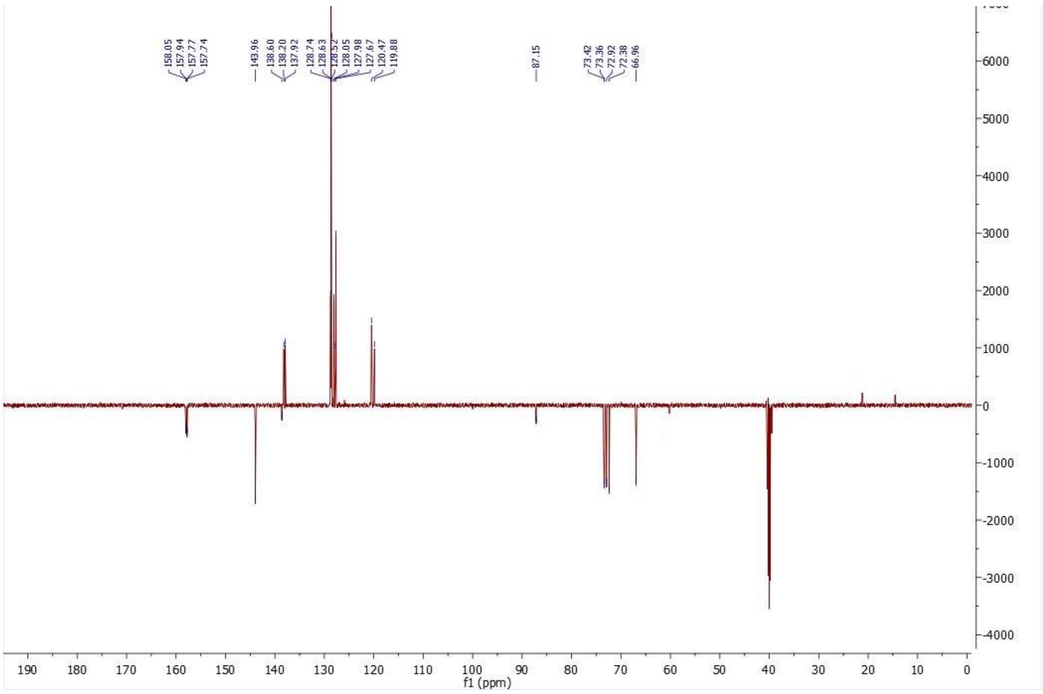

Figure S6. ^{13}C-NMR spectrum of **8** (DMSO-d_6)

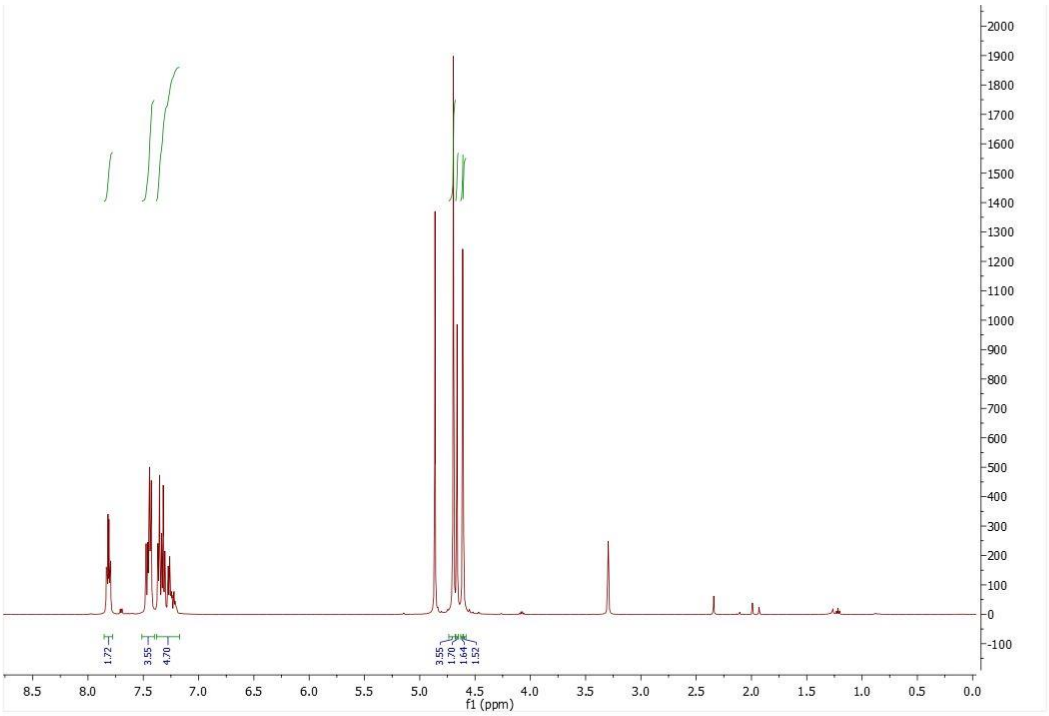

Figure S7. ^{1}H-NMR spectrum of **9** (methanol-d_4)

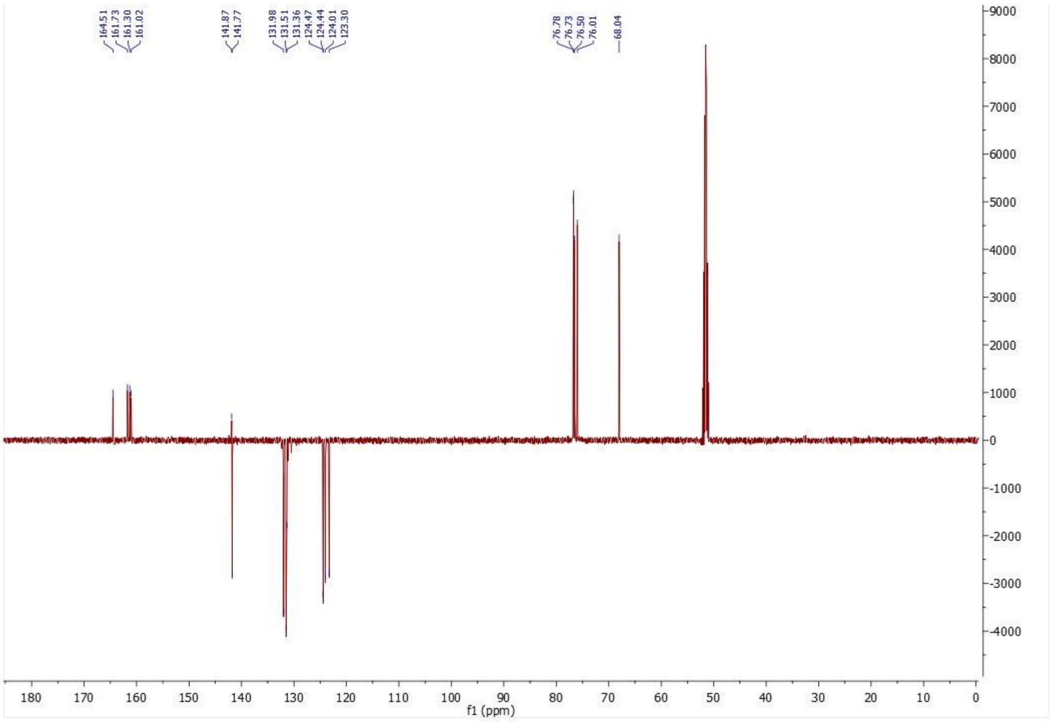

Figure S8. ^{13}C-NMR spectrum of **9** ($CDCl_3$)

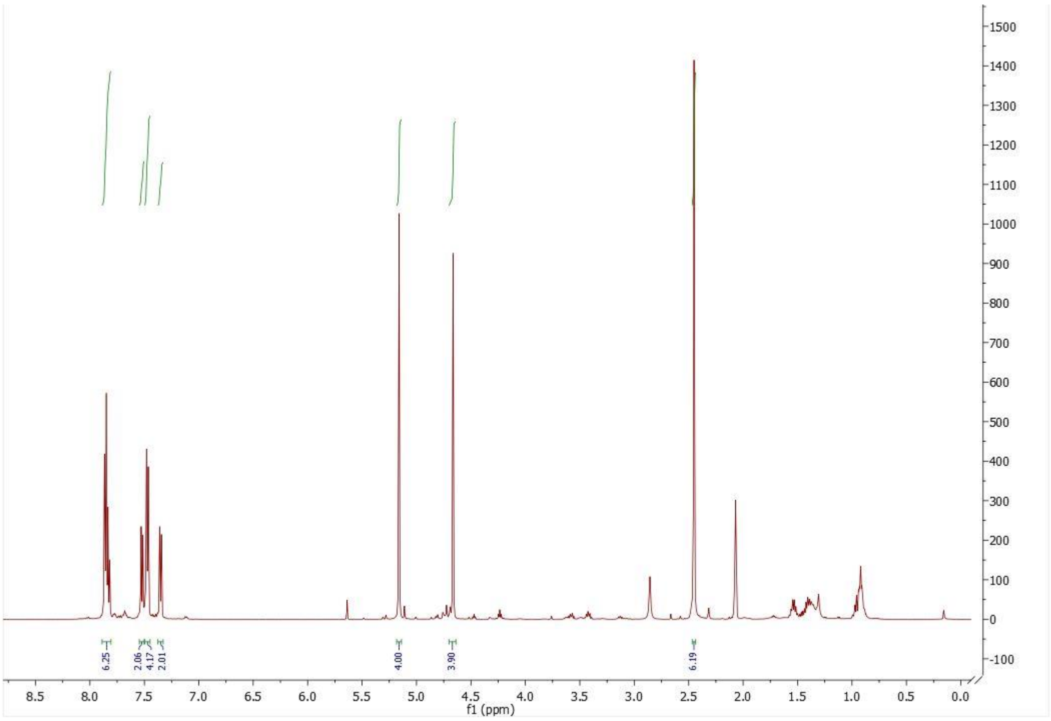

Figure S9. ^{1}H-NMR spectrum of **11** (acetone-d_6)

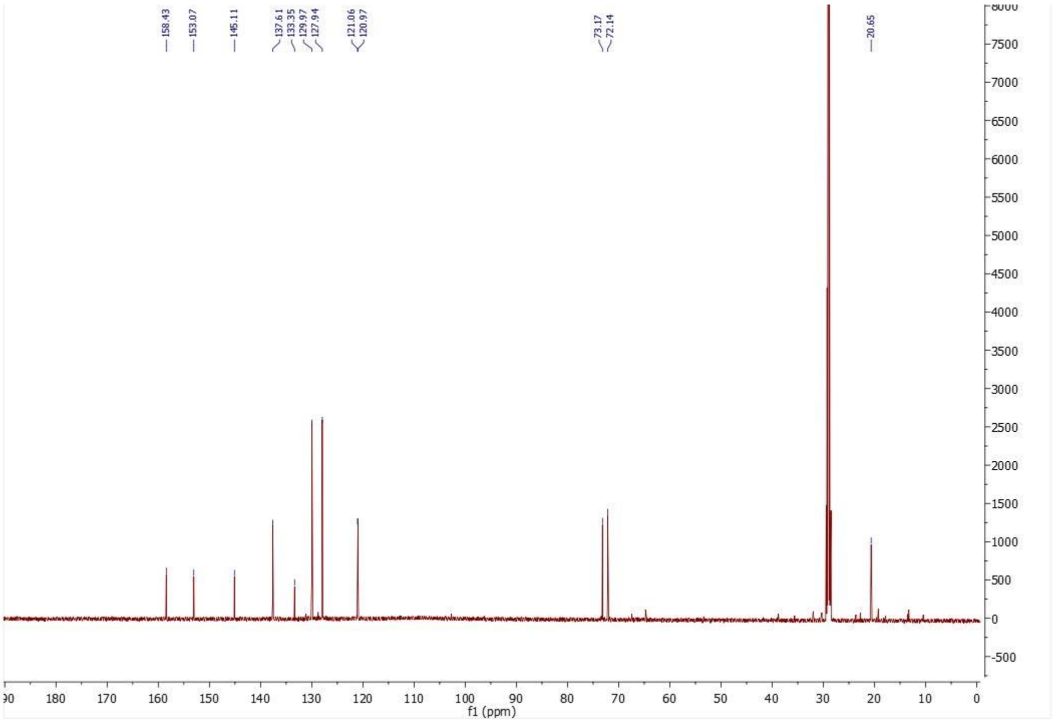

Figure S10. ^{13}C-NMR spectrum of **11** (acetone-d_6)

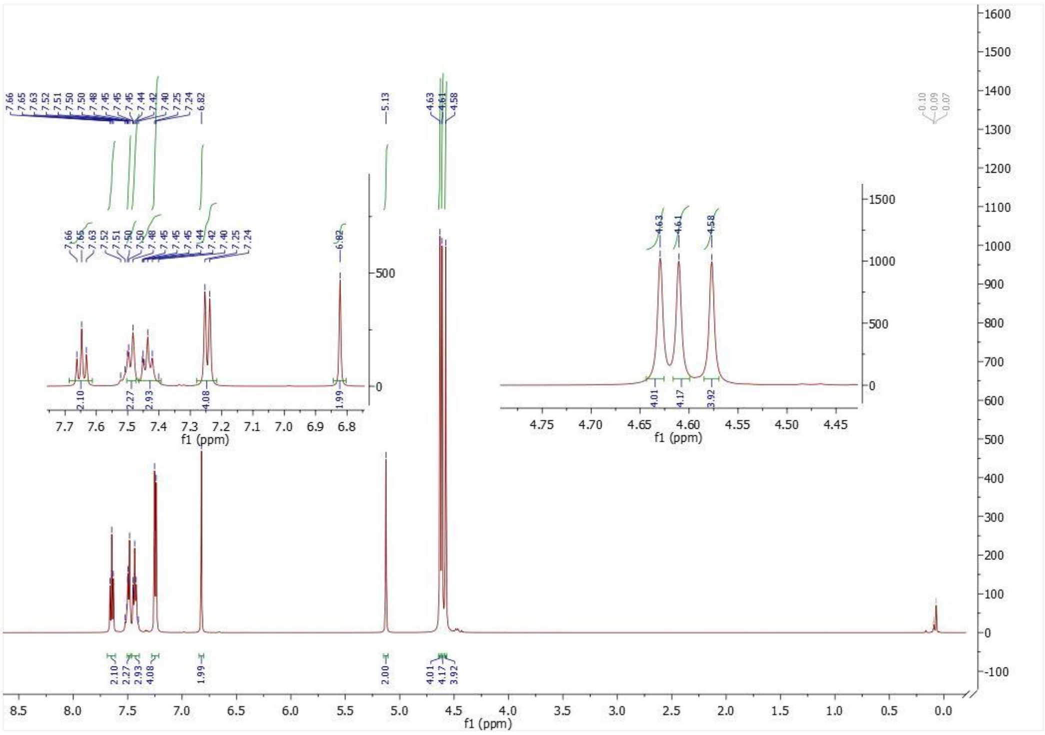

Figure S11. ^{1}H-NMR spectrum of **1** (acetone-d_6)

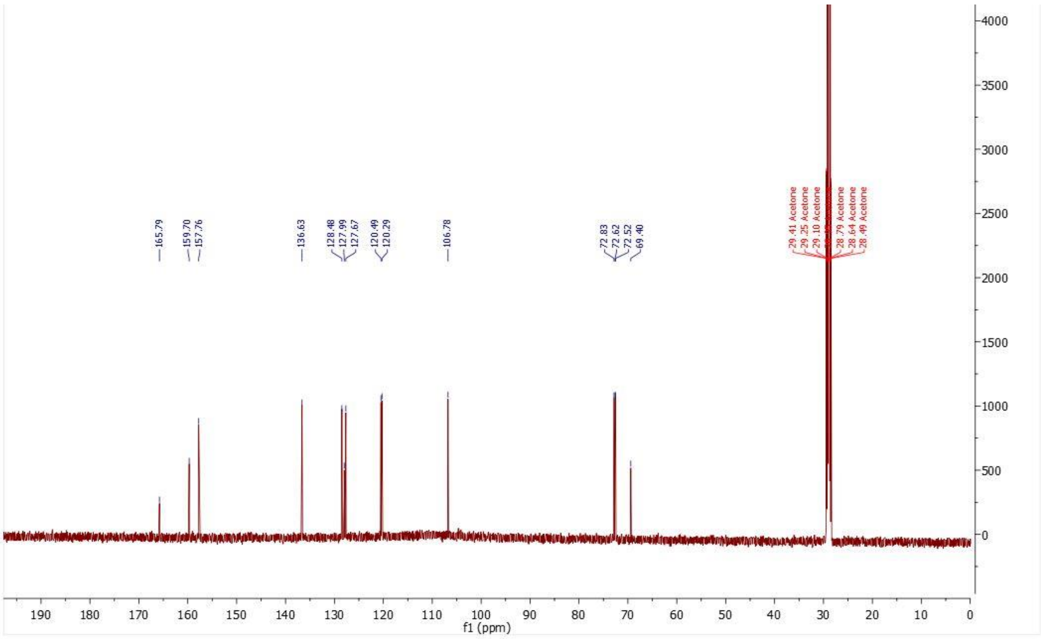

Figure S12. ^{13}C-NMR spectrum of **1** (acetone-d_6)

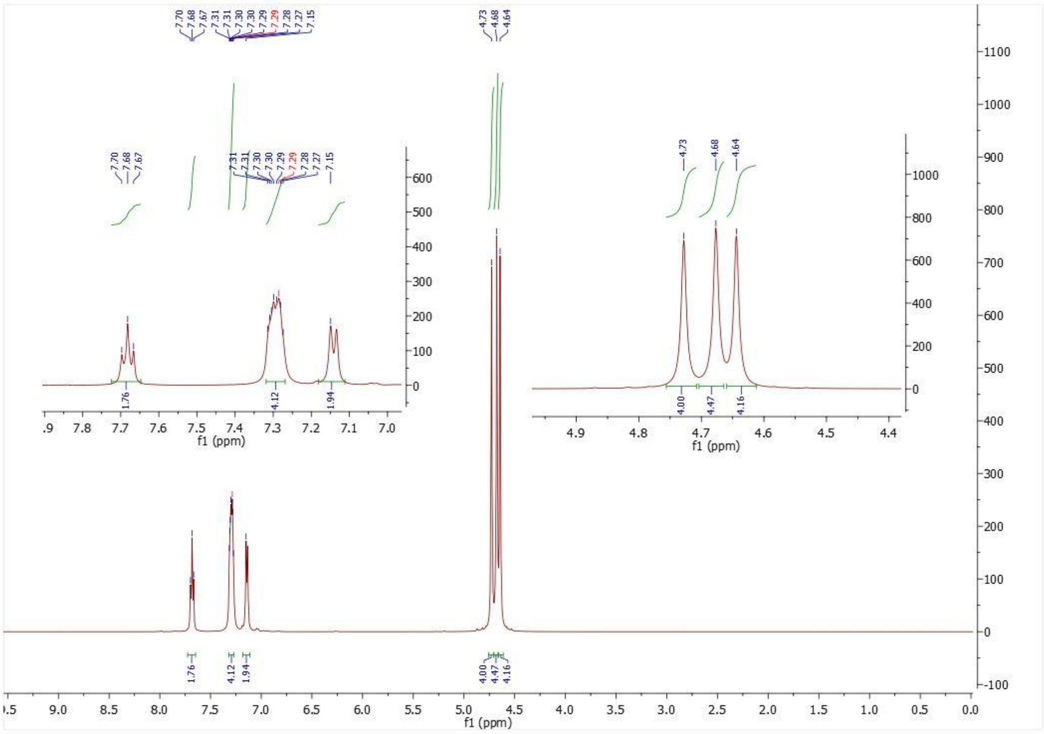

Figure S13. ^{1}H-NMR spectrum of **14** ($CDCl_3$)

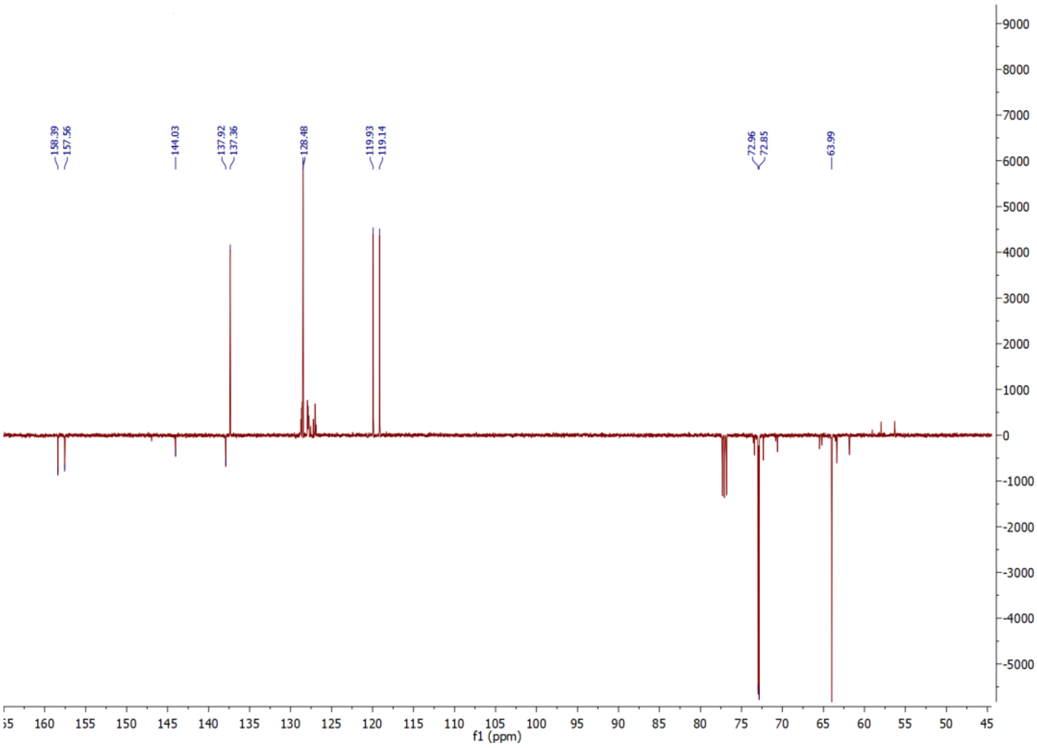

Figure S14. ^{13}C-NMR spectrum of **14** ($CDCl_3$)

2. ^{1}H-NMR and ^{13}C-NMR spectra of the previously reported partially characterized compound 10

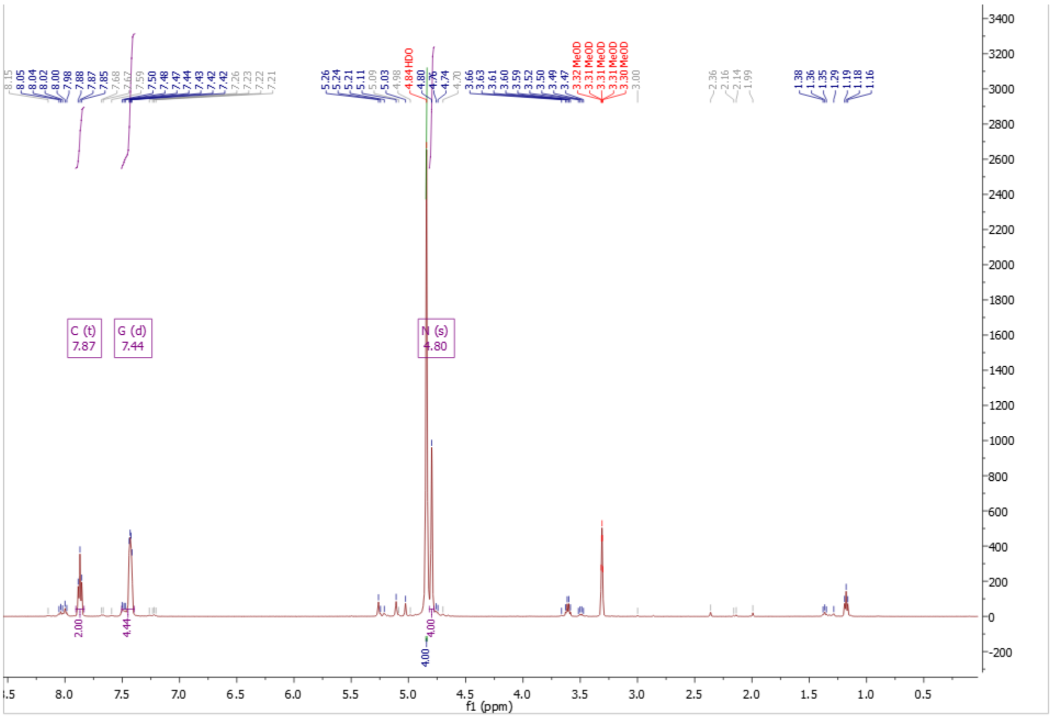

Figure S15. ^{1}H-NMR spectrum of **10** (methanol-d_4)

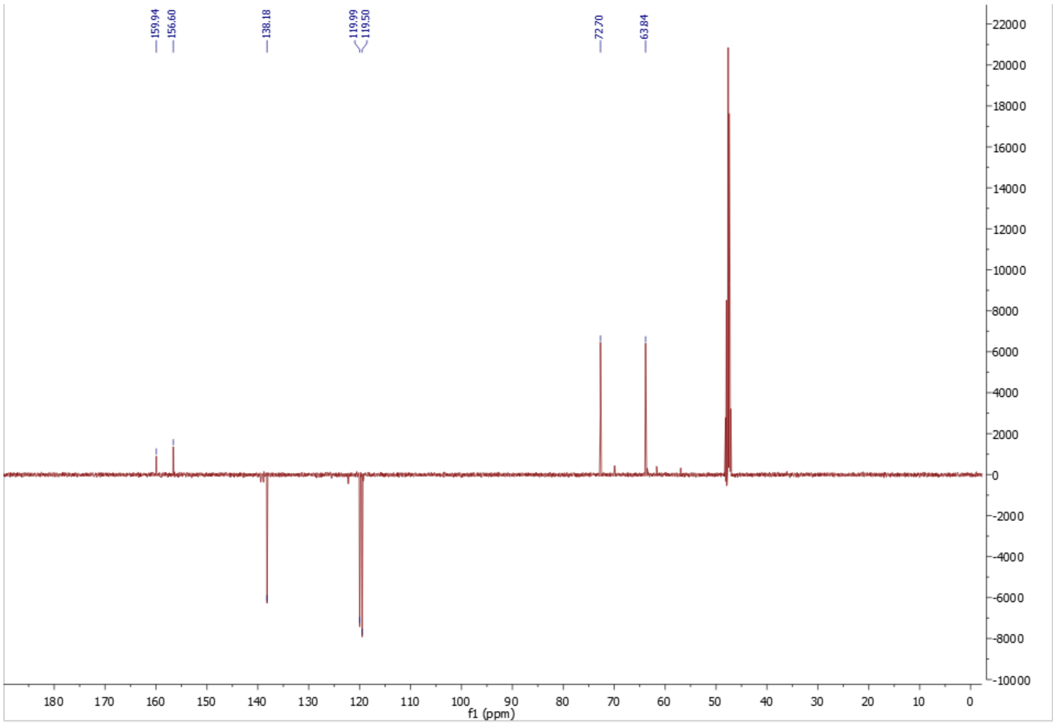

Figure S16. ^{13}C-NMR spectrum of **10** (methanol-d_4)

3. Spectrophotometric investigation of the new crown ether (1)

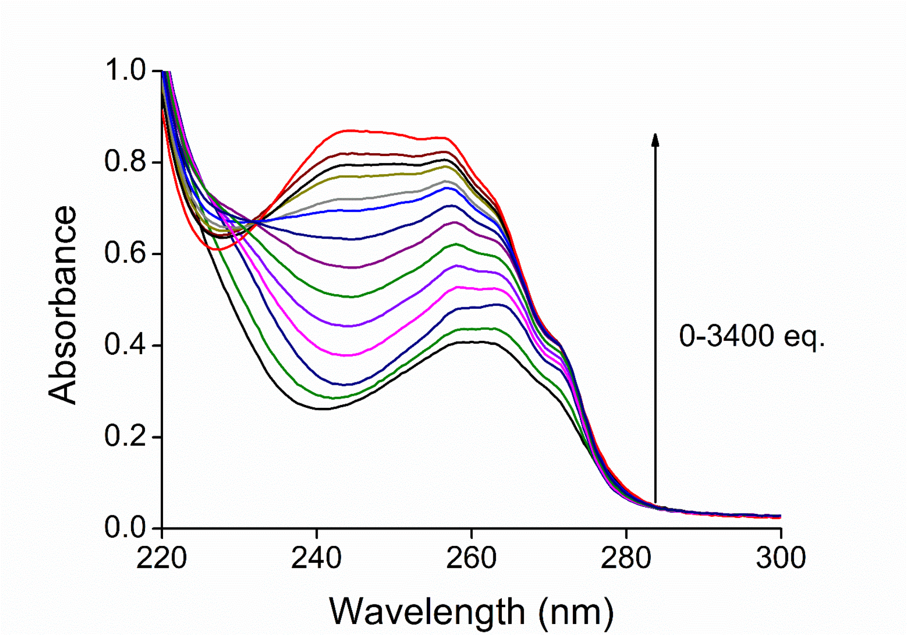

Figure S17. Series of absorption spectra for titrating macrocycle **1** with protonated amine **15** in acetonitrile ($c_{macrocycle}$=5·10^{-7} mol·L^{-1})

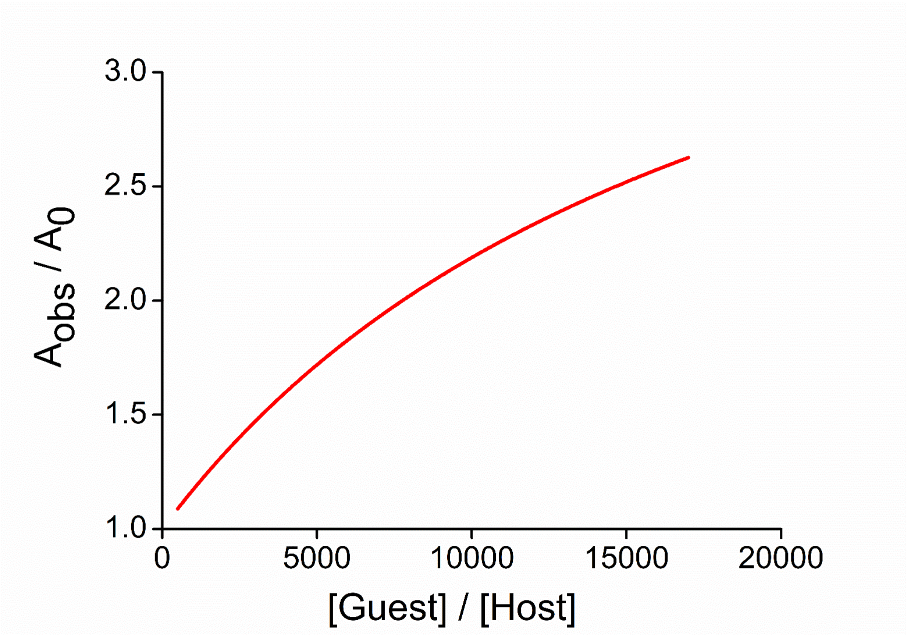

Figure S18. The globally fitted nonlinear regression curve for determining log*K* value based on titrating macrocycle **1** with protonated amine **15** in acetonitrile ($c_{macrocycle}$=5·10^{-7} mol·L^{-1})

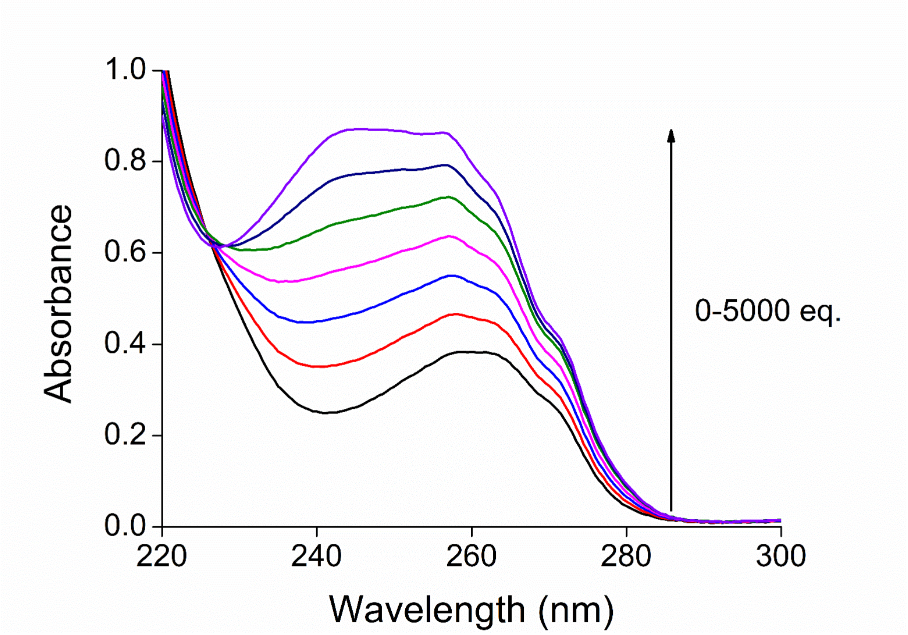

Figure S19. Series of absorption spectra for titrating macrocycle **1** with protonated amine **16** in acetonitrile ($c_{macrocycle}$=5·10^{-7} mol·L^{-1})

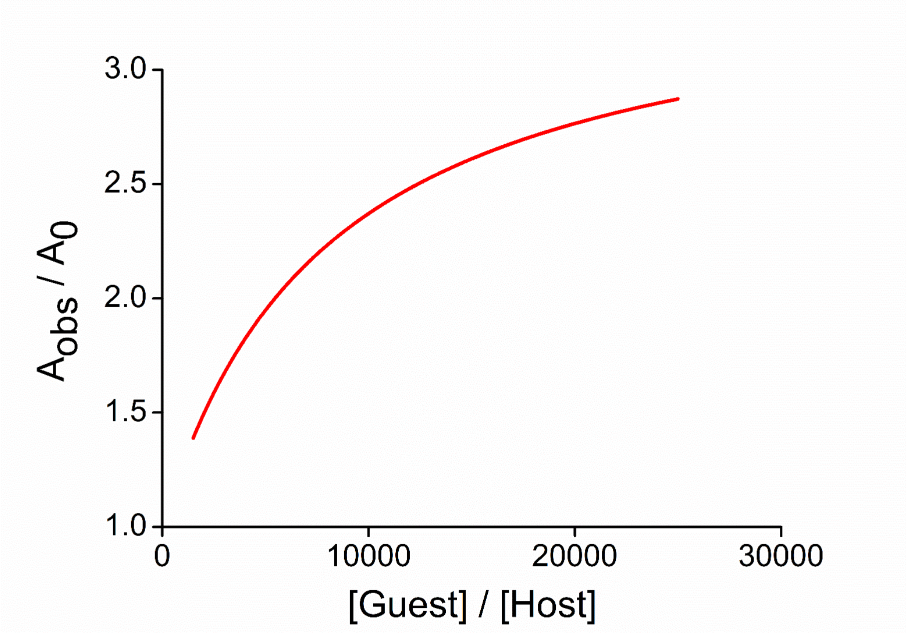

Figure S20. The globally fitted nonlinear regression curve for determining log*K* value based on titrating macrocycle **1** with protonated amine **16** in acetonitrile ($c_{macrocycle}$=5·10^{-7} mol·L^{-1})

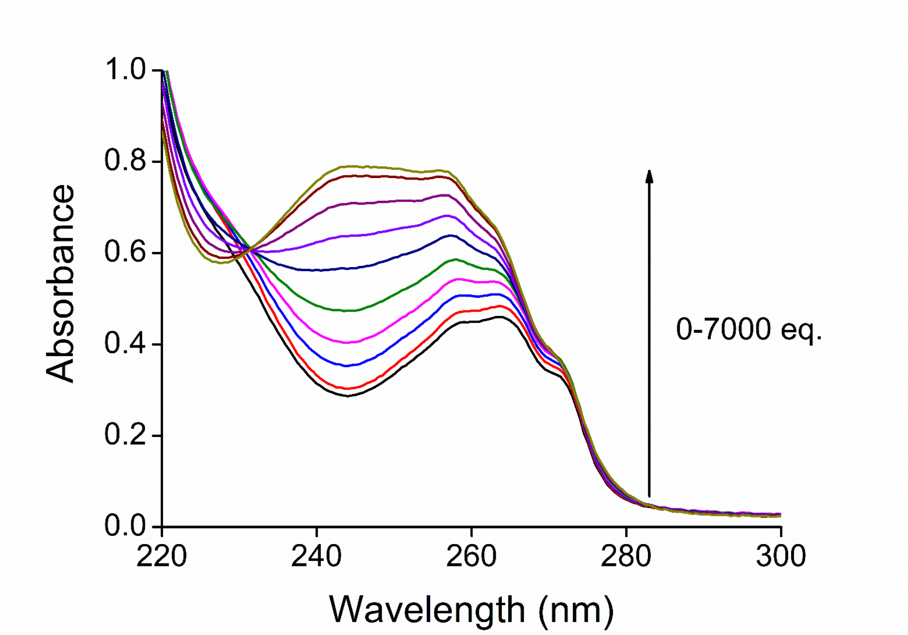

Figure S21. Series of absorption spectra for titrating macrocycle **1** with protonated amine **17** in acetonitrile ($c_{macrocycle}$=5·10^{-7} mol·L^{-1})

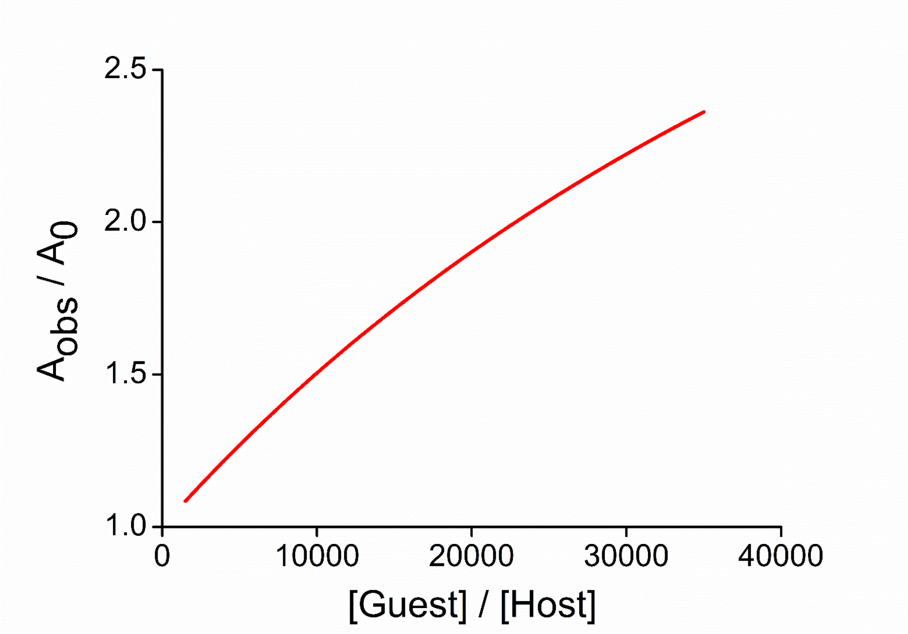

Figure S22. The globally fitted nonlinear regression curve for determining log*K* value based on titrating macrocycle **1** with protonated amine **17** in acetonitrile ($c_{macrocycle}$=5·10^{-7} mol·L^{-1})

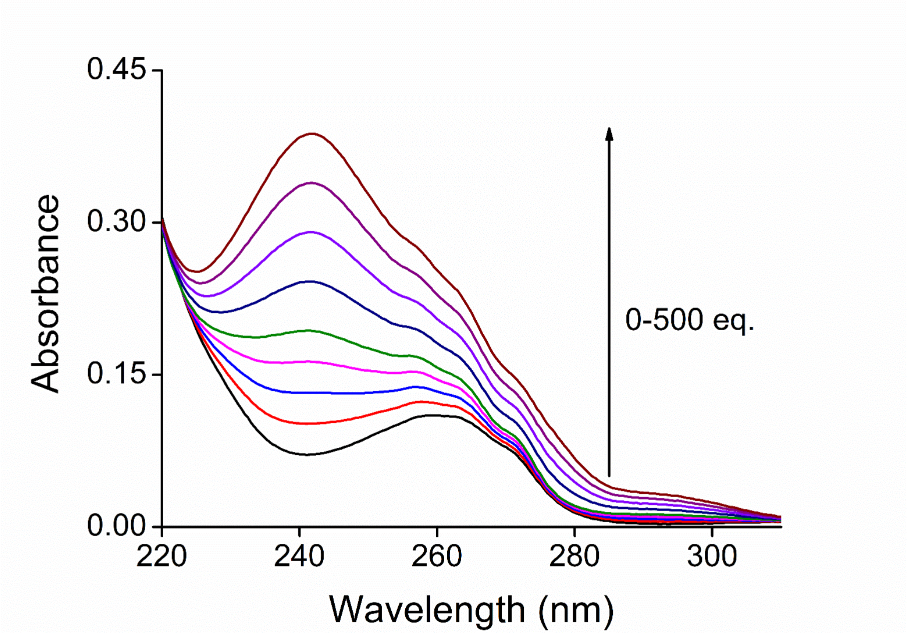

Figure S23. Series of absorption spectra for titrating macrocycle **1** with protonated amine **18** in acetonitrile ($c_{macrocycle}$=5·10^{-8} mol·L^{-1})

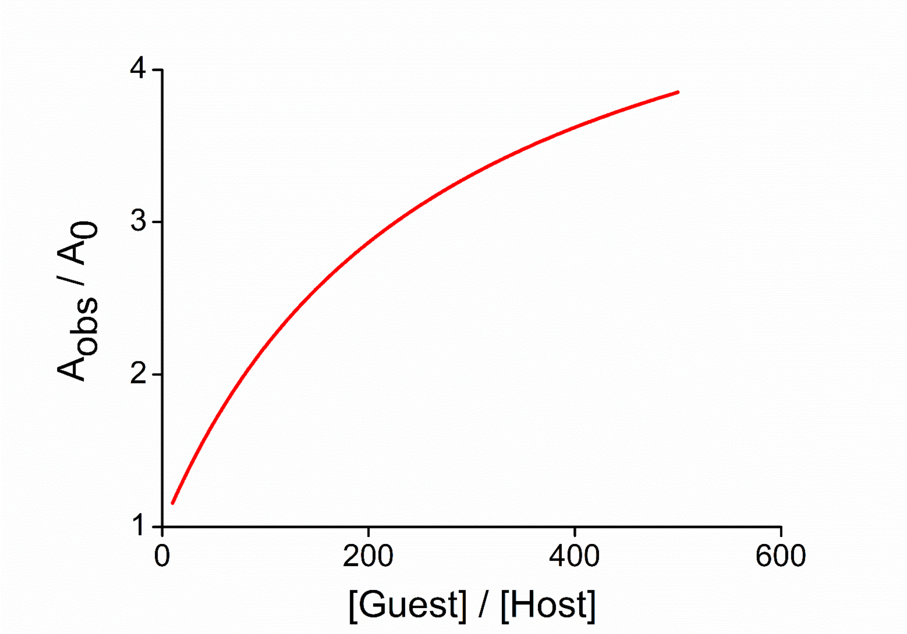

Figure S24. The globally fitted nonlinear regression curve for determining log*K* value based on titrating macrocycle **1** with protonated amine **18** in acetonitrile ($c_{macrocycle}$=5·10^{-8} mol·L^{-1})

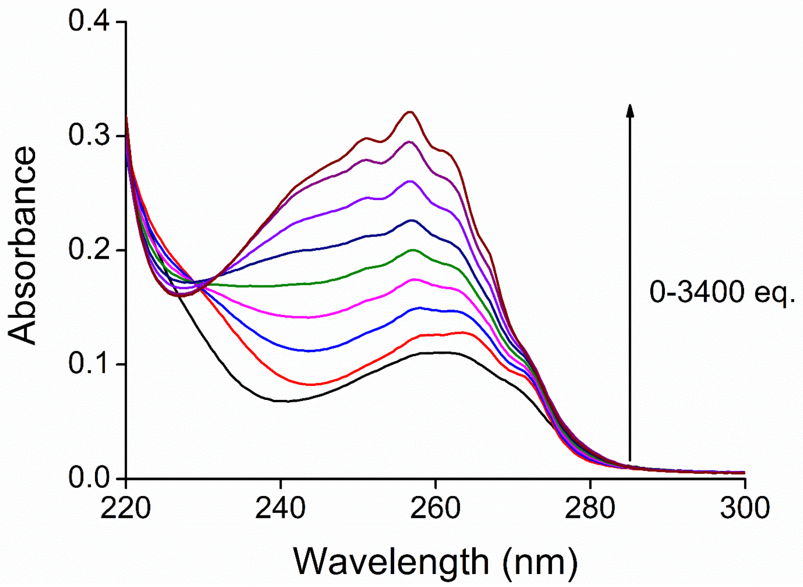

Figure S25. Series of absorption spectra for titrating macrocycle **1** with protonated amine **21** in acetonitrile ($c_{macrocycle}$=5·10^{-8} mol·L^{-1})

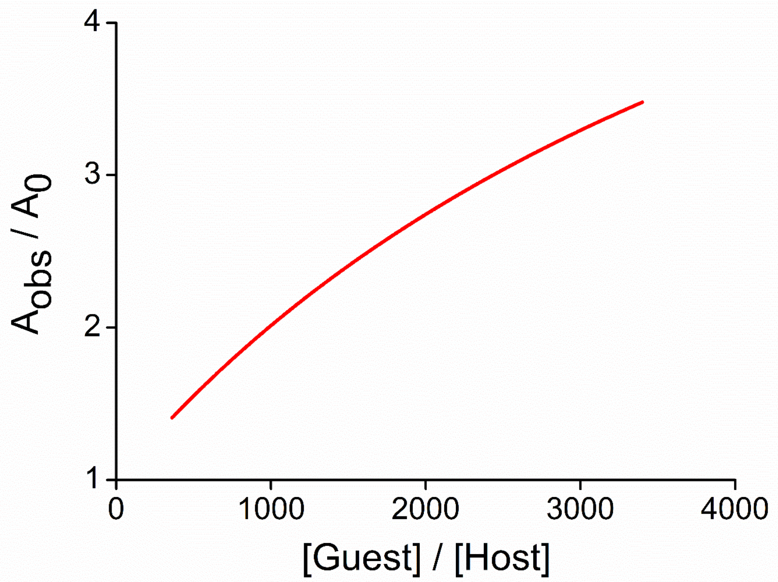

Figure S26. The globally fitted nonlinear regression curve for determining log$K$ value based on titrating macrocycle **1** with protonated amine **21** in acetonitrile ($c_{macrocycle}$=5·10^{-8} mol·L^{-1})

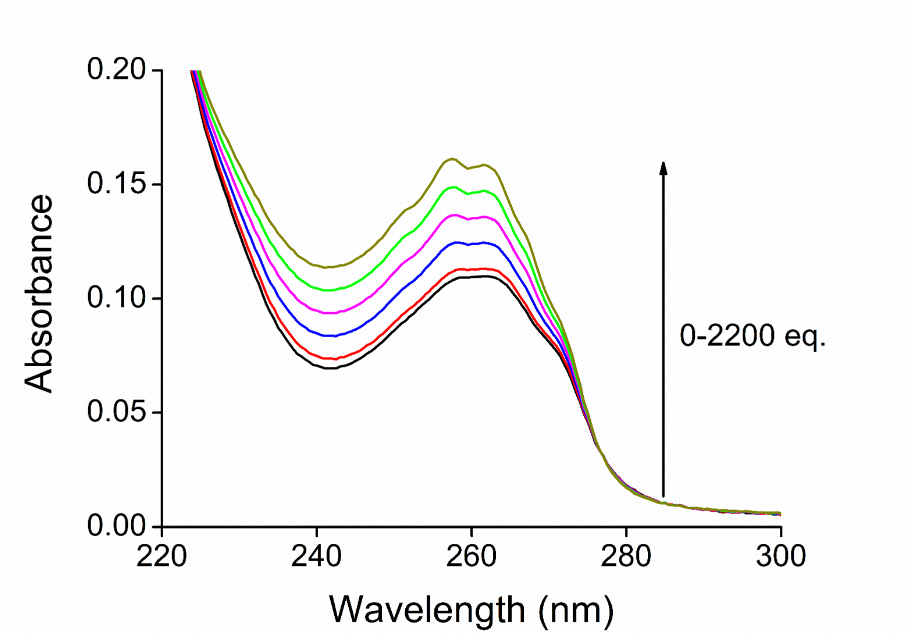

Figure S27. Series of absorption spectra for titrating macrocycle **1** with protonated amine **22** in acetonitrile ($c_{macrocycle}$=5·10^{-8} mol·L^{-1})

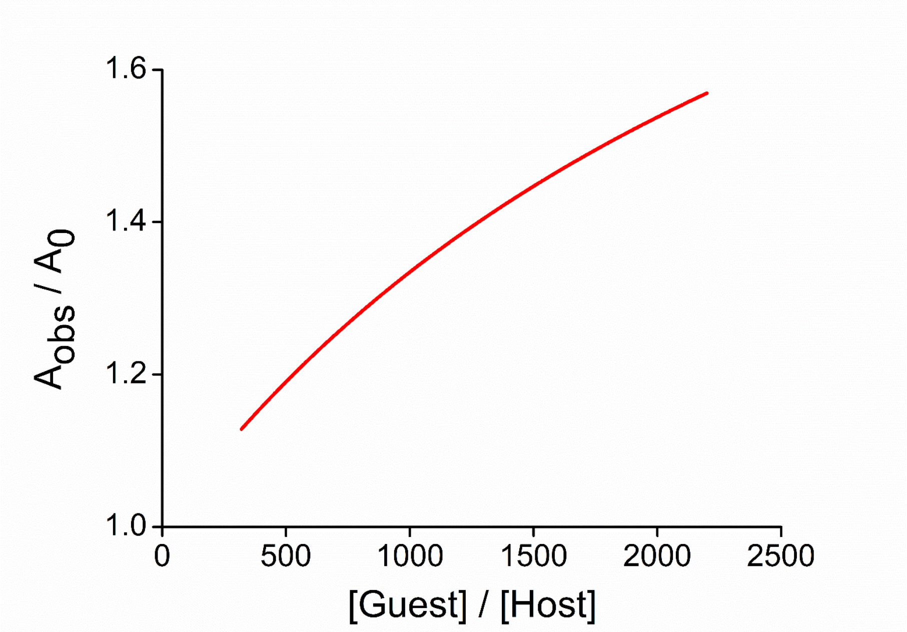

Figure S28. The globally fitted nonlinear regression curve for determining log$K$ value based on titrating macrocycle **1** with protonated amine **22** in acetonitrile ($c_{macrocycle}$=5·10^{-8} mol·L^{-1})

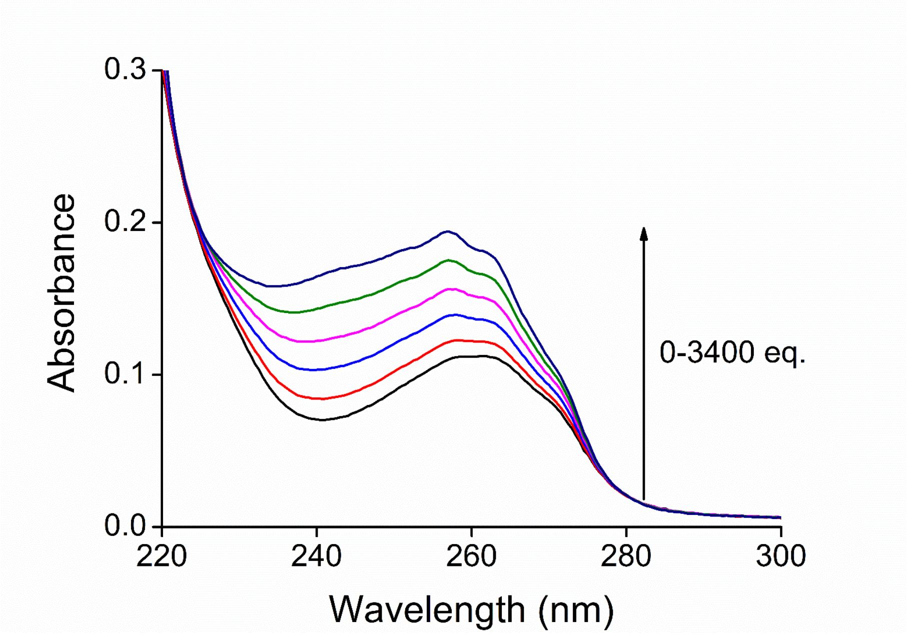

Figure S29. Series of absorption spectra for titrating macrocycle **1** with protonated amine **23** in acetonitrile ($c_{macrocycle}$=5·10^{-8} mol·L^{-1})

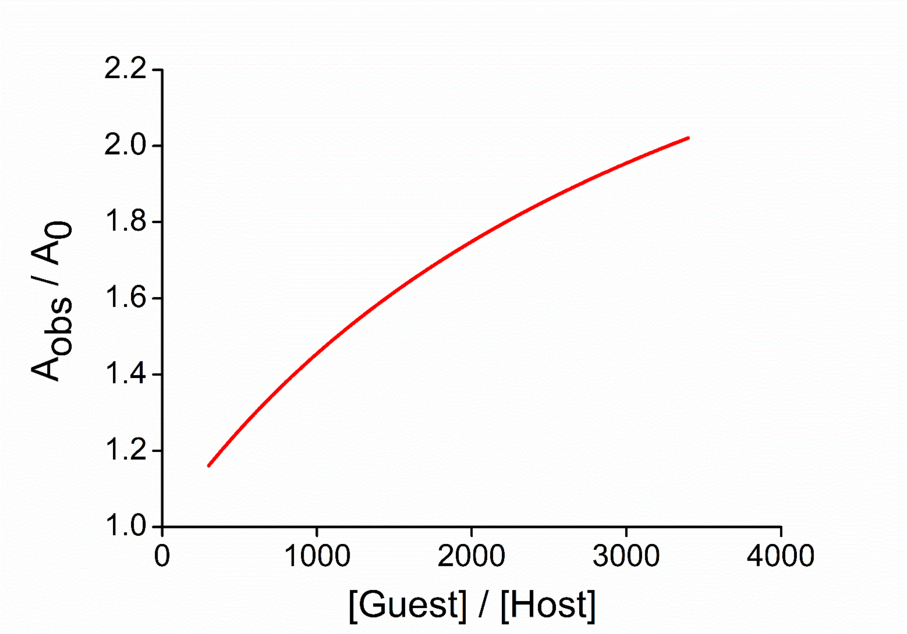

Figure S30. The globally fitted nonlinear regression curve for determining log*K* value based on titrating macrocycle **1** with protonated amine **23** in acetonitrile ($c_{macrocycle}$=5·10^{-8} mol·L^{-1})

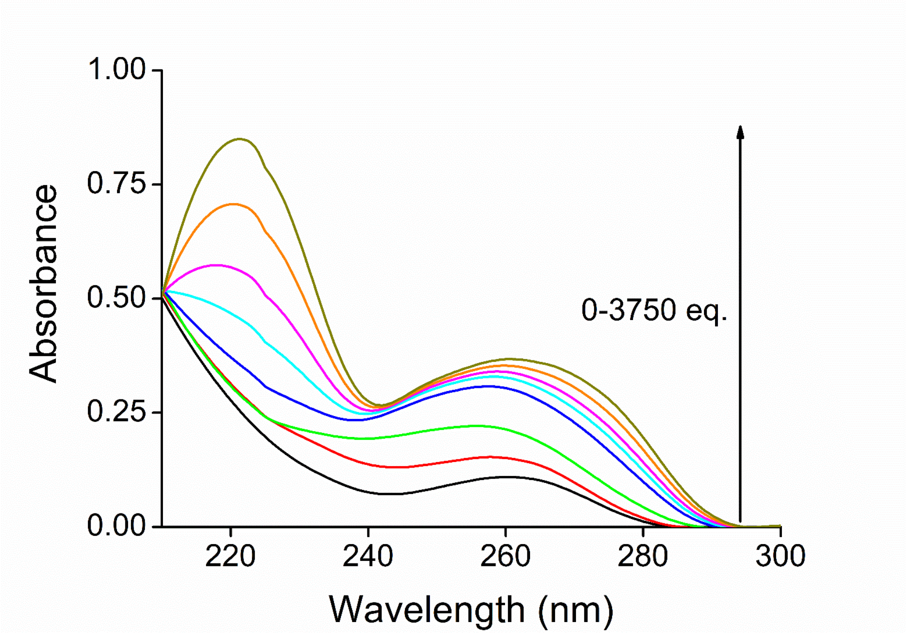

Figure S31. Series of absorption spectra for titrating macrocycle **1** with protonated amine **24** in acetonitrile ($c_{macrocycle}$=5·10^{-8} mol·L^{-1})

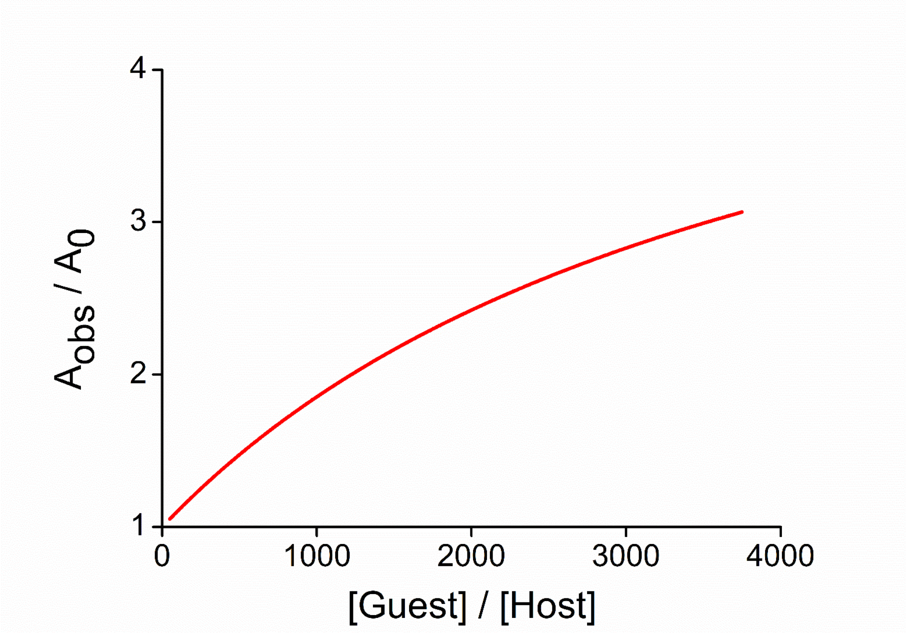

Figure S32. The globally fitted nonlinear regression curve for determining log*K* value based on titrating macrocycle **1** with protonated amine **24** in acetonitrile ($c_{macrocycle}$=5·10^{-8} mol·L^{-1})

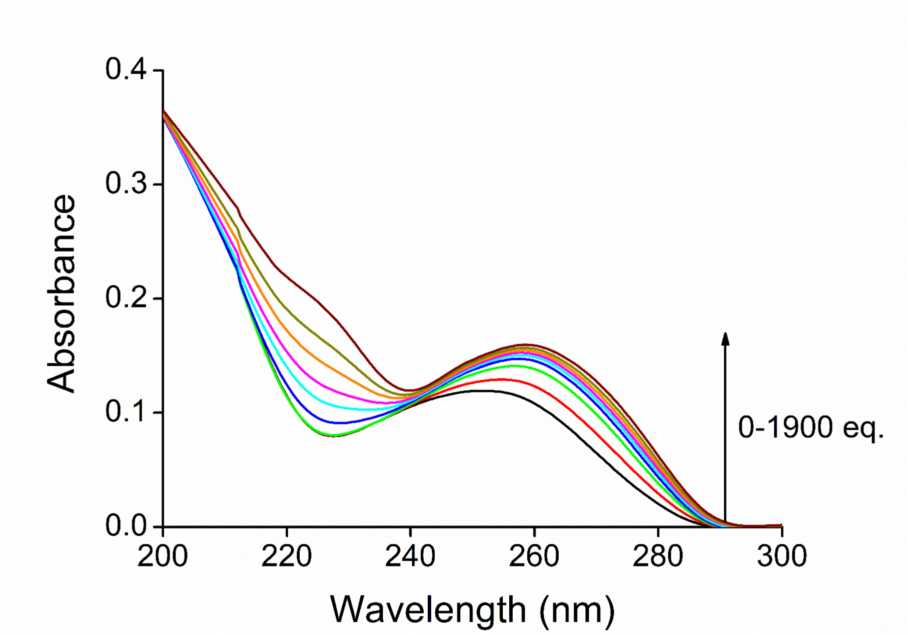

Figure S33. Series of absorption spectra for titrating macrocycle **1** with protonated amine **25** in acetonitrile ($c_{macrocycle}$=5·10^{-8} mol·L^{-1})

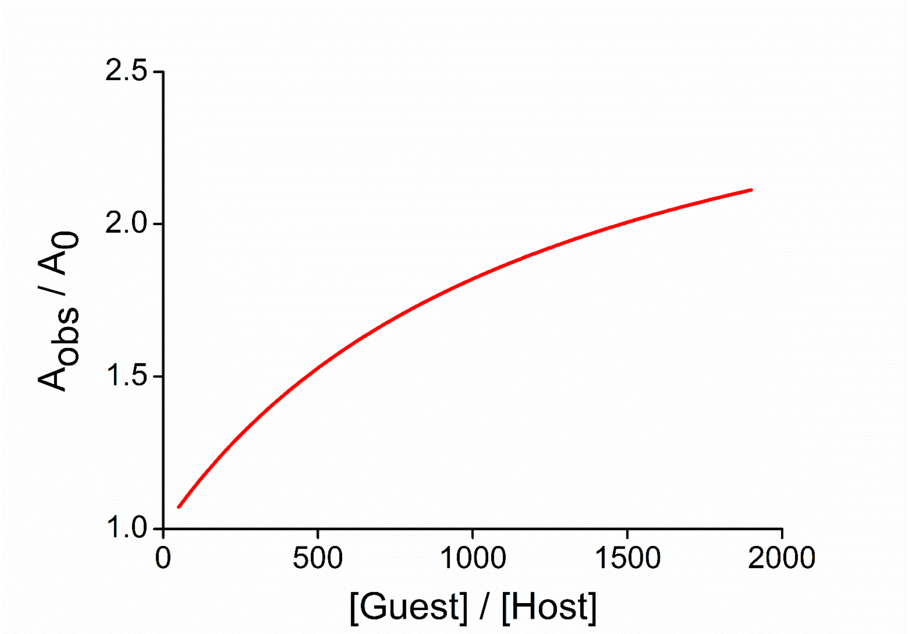

Figure S34. The globally fitted nonlinear regression curve for determining log*K* value based on titrating macrocycle **1** with protonated amine **25** in acetonitrile ($c_{macrocycle}$=5·10^{-8} mol·L^{-1})

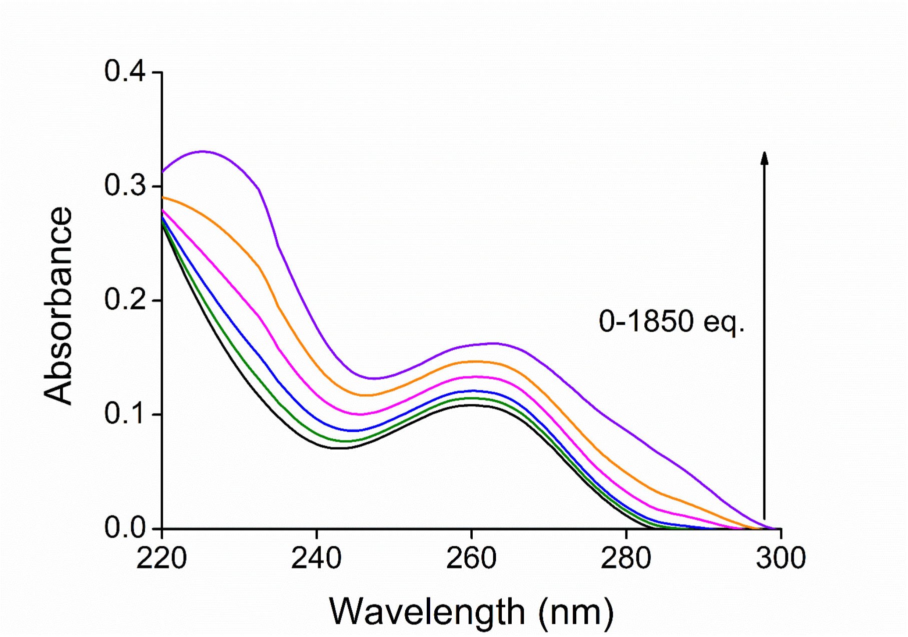

Figure S35. Series of absorption spectra for titrating macrocycle **1** with protonated amine **26** in acetonitrile ($c_{macrocycle}=5\cdot10^{-8}$ mol·L^{-1})

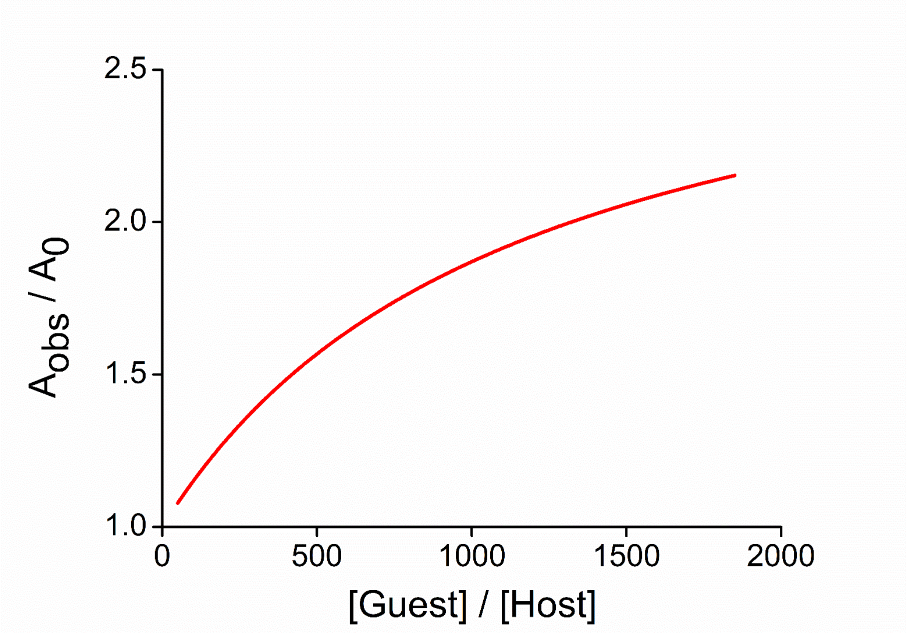

Figure S36. The globally fitted nonlinear regression curve for determining log*K* value based on titrating macrocycle **1** with protonated amine **26** in acetonitrile ($c_{macrocycle}=5\cdot10^{-8}$ mol·L^{-1})

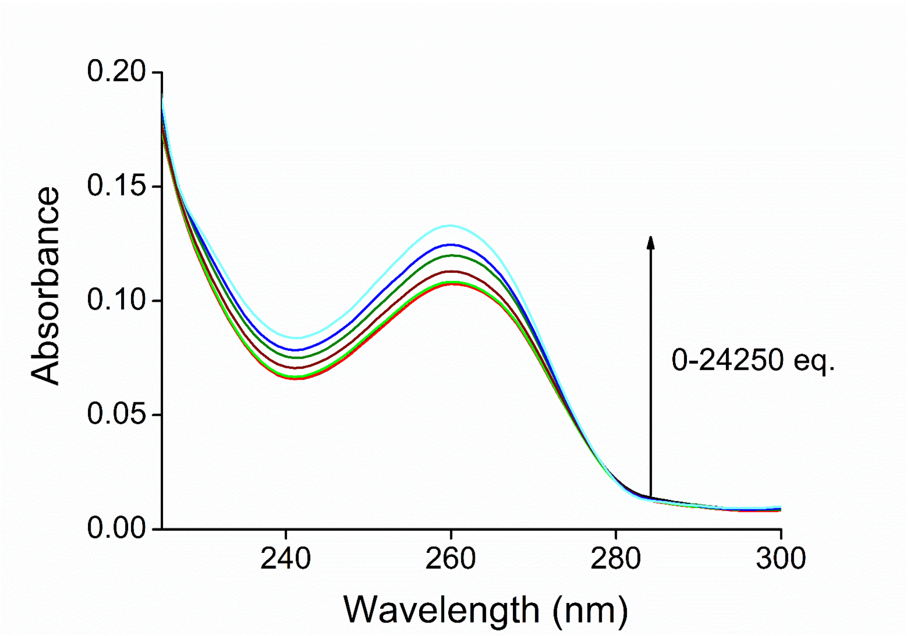

Figure S37. Series of absorption spectra for titrating macrocycle **1** with protonated amine **27** in acetonitrile ($c_{macrocycle}=5\cdot10^{-8}$ mol·L^{-1})

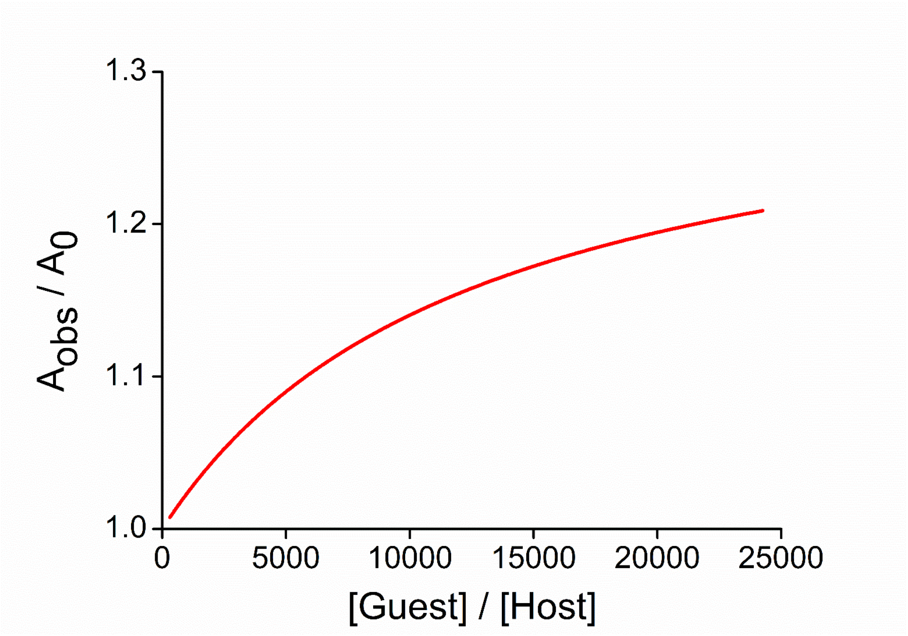

Figure S38. The globally fitted nonlinear regression curve for determining log*K* value based on titrating macrocycle **1** with protonated amine **27** in acetonitrile ($c_{macrocycle}=5\cdot10^{-8}$ mol·L^{-1})
